# Controlled Synthesis of Large Single Crystals of Metal‐Organic Framework CPO‐27‐Ni Prepared by a Modulation Approach: *In situ* Single‐Crystal X‐ray Diffraction Studies

**DOI:** 10.1002/chem.202100528

**Published:** 2021-05-07

**Authors:** Simon M. Vornholt, Caroline G. Elliott, Cameron M. Rice, Samantha E. Russell, Peter J. Kerr, Daniel N. Rainer, Michal Mazur, Mark R. Warren, Paul S. Wheatley, Russell E. Morris

**Affiliations:** ^1^ University of St Andrews North Haugh KY16 9ST St Andrews United Kingdom; ^2^ Department of Physical and Macromolecular Chemistry Faculty of Sciences Charles University Hlavova 8 128 43 Prague 2 Czech Republic; ^3^ Diamond Light Source Harwell Science and Innovation Campus Didcot OX11 0DE United Kingdom

**Keywords:** CPO-27-M, gas adsorption, metal-organic frameworks, modulation synthesis, MOF-74, single crystals

## Abstract

The size of single crystals of the metal‐organic framework CPO‐27‐Ni was incrementally increased through a series of modulated syntheses. A novel linker modulated synthesis using 2,5‐dihydroxyterephthalic acid and the isomeric ligand 4,6‐dihydroxyisophthalic acid yielded large single crystals of CPO‐27‐Ni (∼70 μm). All materials were shown to have high crystallinity and phase purity through powder X‐ray diffraction, electron microscopy methods, thermogravimetry, and compositional analysis. For the first time single‐crystal structure analyses were carried out on CPO‐27‐Ni. High BET surface areas and nitric oxide (NO) release efficiencies were recorded for all materials. Large single crystals of CPO‐27‐Ni showed a prolonged NO release and proved suitable for *in situ* single‐crystal diffraction experiments to follow the NO adsorption. An efficient activation protocol was developed, leading to a dehydrated structure after just 4 h, which subsequently was NO‐loaded, leading to a first NO loaded single‐crystal structural model of CPO‐27‐Ni.

## Graphical abstract

A novel mixed linker modulation approach yielded large single crystals of CPO‐27‐Ni, an analogue that has not yet been able to be studied via single‐crystal X‐ray diffraction due to crystal size limitations. This study investigates the nitric oxide, an important therapeutic agent, gas loading behavior of CPO‐27‐Ni single crystals utilizing an *in situ* gas cell setup using synchrotron radiation.

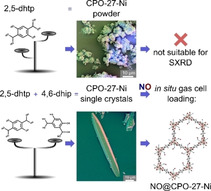

## Introduction

Metal‐organic frameworks (MOFs) are a class of nanoporous materials. They are generally composed of inorganic metal clusters connected through organic polydentate linkers, forming networks with remarkably high porosity.[^1^, ^2^] The almost infinite combination of various linkers and metal clusters unlocks a range of functionalities in these frameworks. Porosity paired with functionality opens a wide field of potential applications for MOFs, including battery materials,[^3^, ^4^, ^5^] catalysis,[^6^, ^7^, ^8^, ^9^] gas separation[^10^, ^11^, ^12^, ^13^, ^14^] and storage,[^15^, ^16^, ^17^, ^18^, ^19^] as well as biomedical applications.[^20^, ^21^, ^22^, ^23^, ^24^] The majority of these applications rely on guest‐host interactions. Guest molecules can enter the framework through the well‐defined pores, and either be trapped in the pore system by weak interactions or by the formation of coordinative bonds with the framework. The latter is often observed in MOFs with coordinatively unsaturated metal sites (CUSs), where a highly reactive metal site is created upon removal of the solvent molecules; we refer to it as activation of the framework. CPO‐27‐Ni, also known as Ni‐MOF‐74,^[25]^ has the highest reported density of CUSs making it one of the most studied MOFs,^[26]^ and has proven ability to coordinate gases.[^17^, ^27^, ^28^]

Modulation chemistry is a new approach for the synthesis of MOFs, and strategic implementation in MOF syntheses has been shown to be useful in controlling the crystallite size.^[29]^ Modulation agents are usually molecules that mimic the functionality of the linker, but are monodentate, hence unable to form a continuous network. Brozek and co‐workers performed a comprehensive study on the control of crystallite sizes of common MOFs down to the nanometer scale.^[30]^ Other works have concentrated on the effect of pKa values of different modulators, to control defect formation in UiO‐66.[^31^, ^32^, ^33^] It has also been shown that robust single crystals can be achieved by a modulation approach.^[34]^ Others have focused on a linker modulation to access new phases.

Long and co‐workers have discovered a family of structural isomers of CPO‐27‐M using the competing meta functionalised linker 4,6‐dihydroxyisophthalic acid or *m‐*dioxidobenzene‐dicarboxylic acid (4,6‐dhip or *m*‐dobdc respectively) as opposed to the traditional 2,5‐dihydroxyterephthalic acid (2,5‐dhtp).^[35]^ Both linkers can be used individually to synthesise functional MOF frameworks,[^19^, ^35^, ^36^] but have never been used together in a modulated synthesis to gain control over the crystallite size of one phase. These linkers can be synthesised via a simple Kolbe‐Schmitt reaction from their readily available precursors, resorcinol (for 4,6‐dhip) or hydroquinone (for 2,5‐dhtp), in the presence of KHCO_3_.^[35]^ Our group has recently published a modulated synthesis to gain access to single crystals of CPO‐27‐M (M=Zn, Mg).^[37]^ Single crystals of CPO‐27‐Ni, large enough for single‐crystal X‐ray diffraction (SXRD) analysis, however, have always been elusive but are crucially needed to elucidate the intrinsic adsorption and guest‐host properties of the framework with high accuracy.^[38]^ In this study we report a novel mixed linker modulation approach using both 2,5‐dhtp and the competing linker 4,6‐dhip for the synthesis of large single crystals of CPO‐27‐Ni. For the first time, single crystals of CPO‐27‐Ni are available that are suitable for *in situ* SXRD gas adsorption of biologically important nitric oxide (NO).

NO is a diatomic, radical gas which is toxic in high concentrations and a known pollutant created during the combustion of fuels. Porous materials have been a means for the removal of NO from the atmosphere.^[39]^ Despite its toxicity, NO has gained much attention in recent years as a potential therapeutic agent, which can display potent antimicrobial,[^23^, ^24^] vasodilatory,[^22^, ^40^, ^41^] and wound healing effects when administered within an appropriately low concentration threshold.[^22^, ^40^, ^41^] The ability to harness the beneficial effects of NO rely on local administration in controlled concentration thresholds; however, due to its gaseous nature and reactivity with oxygen this has proved difficult.[^16^, ^22^, ^23^, ^42^] MOFs have been investigated as NO delivery agents due to their ability of controlled gas storage and release,[^42^, ^43^] however in order to fully understand this process, a mechanistic study is needed. The local structure of CPO‐27‐Ni upon NO coordination has previously been studied via Rietveld and spectroscopic methods.[^43^, ^44^, ^45^] Furthermore, *in situ* gas adsorption studies involving a series of different guest molecules were carried out on single crystals of CPO‐27‐Co.^[46]^ Until now, the synthesis of CPO‐27‐Ni single crystals large enough for a SXRD study proved challenging. As such a NO adsorption study on CPO‐27‐Ni, the analogue with the most promising NO storage and release properties, was yet to be conducted.

## Experimental Methods and Materials

A detailed procedure of the synthesis of all materials, further discussion about the slight crystallographic differences between **v** and **vi**, and results of LeBail refinements, compositional and thermogravimetric analyses (**i**–**vi**) are provided in the Supporting Information. Furthermore, a full description of employed analyses and underpinning crystallographic tables can be found.

## Results and Discussion

### Synthesis and Characterisation of CPO‐27‐Ni Single Crystals

**Figure 1:**
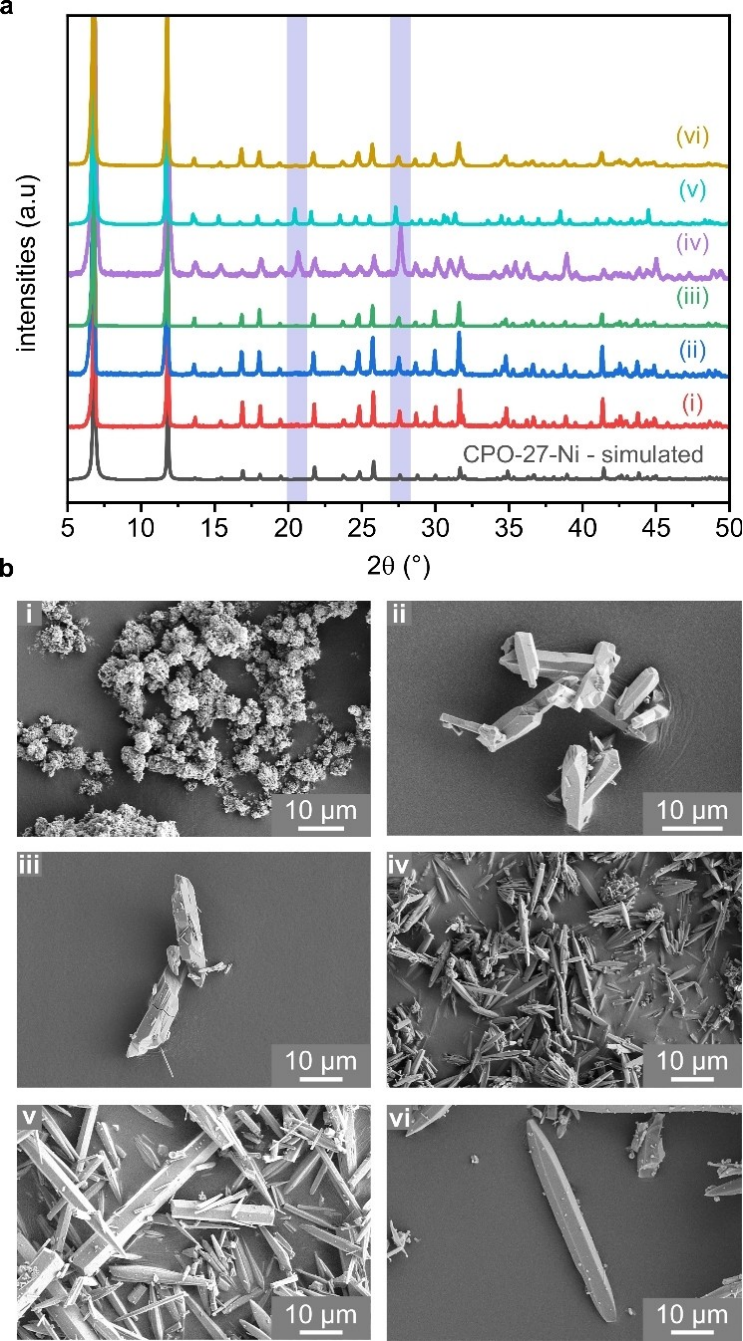
Characterisation of CPO‐27‐Ni materials and the isostructural MOF M‐4,6‐dhip (M=Co, Ni). Slight structural differences that lead to increased intensity for 4,6‐dhip materials are highlighted in purple. a) PXRD patterns of synthesised materials, compared to a simulated pattern of CPO‐27‐Ni (grey) and Co‐4,6‐dhip (v). b) SEM micrographs of crystallites. i) CPO‐27‐Ni from a stoichiometric synthesis, ii) small single crystals of CPO‐27‐Ni, iii) medium sized single crystals CPO‐27‐Ni, iv) small single crystals of Ni‐4,6‐dhip, v) single crystals of Co‐4,6‐dhip, vi) large single crystals of CPO‐27‐Ni.

The CPO‐27‐M (M=Co, Fe, Mn, Mg, Ni, Zn) framework, with a structure formula of [M_2_(ligand) ⋅ (H_2_O)_2_] ⋅ 8H_2_O, is extensively described in the literature with many different synthesis routes reported: from solvothermal DMF^[25]^ or THF^[47]^ based methods to room temperature, water‐based methods^[61]^ for scale‐up purposes. Also of interest, Kapelewski and co‐workers^[35]^ have identified a structural isomer of the CPO‐27‐M family when the isomeric linker 4,6‐dihydroxyisophthalic acid (4,6‐dhip) was used. However, none of these methods have ever produced crystals of CPO‐27‐Ni large enough for SXRD analysis.[^25^, ^35^, ^37^, ^47^, ^48^, ^49^] In this present work we employed both a solvothermal (THF/water) and hydrothermal (water) based approach to grow single crystals of CPO‐27‐Ni; an analogue of this family that has not yet been reported to crystallise as large single crystals. Figure 1 displays the PXRD patterns (Figure 1a) and the SEM micrographs (Figure 1b) of the synthesised CPO‐27‐Ni materials with increasing crystallite size and their structural isomers using the 4,6‐dhip (M=Co, Ni) ligand; a scheme of the different linkers is visualised in Figure S1 (Supporting Information).

A polycrystalline yellow‐brown powder of CPO‐27‐Ni (**i**) was obtained in good yield (91 % based on Ni) following a stoichiometric, solvothermal procedure.^[47]^ By offering the reaction mixture an excess of 2,5‐dhtp linker (equimolar), the crystallite size of the primary particles could be increased to elongated rods (3–10 μm) as seen in Figure 1b‐**ii**. By comparison with a simulated PXRD pattern, both **i** and **ii** displayed phase‐pure CPO‐27‐Ni. The anion of the nickel salt acts as a weak base and slowly deprotonates the linker as the reaction proceeds. Increasing the amount of linker in the mixture leads to an overall more acidic system and simultaneously decreases the metal ratio relative to the linker. While this type of modulation is in principle a pH controlled approach, which can increase the particle size, it is also a form of ‘starving’ the reaction as a lower amount of metal is offered, compared to a regular, stoichiometric synthesis.[^29^, ^30^]

This may also be described as a form of nucleation control, something that has been observed to enlarge the crystallite size of zeolites in previous studies.^[50]^ The following syntheses were conducted employing the above described modulation approach. The crystallite size of CPO‐27‐Ni was further increased by using a higher reaction temperature (130 °C) and recrystallised 2,5‐dhtp (in‐house synthesis). The 2,5‐dhtp for the reaction of **iii** was synthesised following a Kolbe‐Schmitt reaction approach from hydroquinone.[^35^, ^51^, ^52^] The beige‐brown product was recrystallised, and thin yellow needles formed. Using this linker, medium sized rods (20−30 μm) of CPO‐27‐Ni (Figure 1b‐**iii**) were obtained. Furthermore, the isomeric linker 4,6‐dihydroxyterephthalic acid (4,6‐dhip) could be made from resorcinol and was used without purification for the synthesis of M‐4,6‐dhip (M=Co, Ni), the structural isomers of CPO‐27‐M. Ni‐4,6‐dhip (**iv**) (mint‐green microcrystalline powder) and Co‐4,6‐dhip (**v**) (pink microcrystalline powder) were synthesised. The latter was synthesised by adding four equivalents of benzoic acid, yielding single crystals, whose SXRD structure was used to simulate a powder pattern for the M‐4,6‐dhip framework.

As seen in Figure 1a, reflections for **v** are slightly shifted towards lower 2θ when compared to Ni‐4,6‐dhip (**iv**). This indicates a slightly larger unit cell for Co‐4,6‐dhip; a slightly enlarged unit cell is also seen for CPO‐27‐Co when compared to its nickel analogue.^[46]^ LeBail refinements of the powder patterns (Table S3 and Figure S2) revealed that the difference between the Ni‐4,6‐dhip cell is ∼125 Å^3^ (in comparison to Co‐4,6‐dhip); the obtained unit cells for the CPO‐27‐Ni samples are ∼50 Å^3^ smaller than the unit cell for CPO‐27‐Co. M‐4,6‐dhip is discussed in the literature to be isostructural to CPO‐27‐Ni.[^19^, ^35^] However, the atoms in the unit cell are oriented slightly differently in some cases. As a result of this, the space group for M‐4,6‐dhip changes to *R*3*m* from *R* $\overset{-}{3}$ (seen for classic CPO‐27 frameworks). Additionally, intensity variations are observed in the respective powder patterns when M‐4,6‐dhip are compared to those of CPO‐27‐M (see highlighted peaks in Figure 1b). M‐4,6‐dhip displays stronger reflections for the (401) (20.44° 2θ) and the (202) planes (27.65° 2θ), respectively. These reflections correspond to the arrangement of the linker in M‐4,6‐dhip while fewer atoms are on those planes in the CPO‐27‐Ni framework, which therefore results in lower intensities. Other intensity differences are given for the corresponding planes (3$\overset{-}{1}$ 1), (5$\overset{-}{3}\overset{-}{1}$ ), and (6$\overset{-}{5}\overset{-}{1}$ ), where higher intensities for the CPO‐27‐Ni framework are observed. To indicate the (minor) structural differences between these isomers, the crystal structure models of CPO‐27‐Co (pure 2,5‐dhtp synthesis), CPO‐27‐Ni (**vi** – mixed linker modulation approach), and Co‐4,6‐dhip (pure 4,6‐dhip synthesis) are displayed in Figure S3. Figure S3a visualises the hexagonal pore, where oxygen atoms of the *hydroxy* groups are highlighted in blue for better visualisation. The meta functionality in 4,6‐dhip results in a slightly different coordination with the metal centres, which results in a decreased angle (as shown in Figure S3b). Thus, the sides of the hexagon are slightly bent outwards, and the overall pore shape appears to be rounder compared to CPO‐27.

Both linkers can be used individually to form a MOF, as described above. Previous studies have discussed the concept of a mixed linker synthesis to introduce versatile functionality in one framework; however, a mixed linker approach was not yet employed to control the crystallite size of one desired phase.[^53^, ^54^] Interestingly, following a mixed linker approach with equimolar amounts of 2,5‐dhtp and 4,6‐dhip, large single‐crystal rods (60–70 μm) of CPO‐27‐Ni (Figure 1b‐**vi**) were obtained. Stoichiometric amounts of commercially available 2,5‐dhtp were mixed with another equivalent of 4,6‐dhip and reacted with nickel acetate tetrahydrate. As Figure 1a shows, all CPO‐27‐Ni materials are single phase and pure (within the limits of PXRD),^[55]^ when compared with a simulated pattern. Even the material afforded from a mixed linker approach (**vi**) does not show traces of the competing MOF (Ni‐4,6‐dhip) in the bulk sample. Initial visual confirmation is given as the mint‐green crystals of the competing Ni‐4,6‐dhip phase are not observed in **vi** – a sample with a distinct yellow‐brown colour common for CPO‐27‐Ni. Furthermore, the PXRD pattern (Figure 1a‐**vi**) also only shows CPO‐27‐Ni reflections. Additionally, all materials were subjected to thermogravimetric and elemental analysis. The TGA profiles (Figure S4) show a significant mass loss of ∼30 % in the temperature range of 25–200 °C. This is in excellent agreement with the theoretical mass loss of eight loosely coordinated water molecules, which are trapped in the pore system of the MOF while the two additional water molecules are still bound to the metal centre. This is also reflected by the results of the elemental analysis. All materials were within the expected ranges for [Ni_2_(C_8_H_2_O_6_) ⋅ (H_2_O)_2_] ⋅ 8H_2_O (see Table S4). The materials are stable up to a temperature of 260 °C, after which the framework decomposes.^[56]^

On the basis of SXRD we can rule out significant contribution to the CPO‐27‐Ni framework by 4,6‐dhip. Together with the absence of any irregularities in the thermal and elemental analysis, we infer that the isomeric linker 4,6‐dhip has only a modulating effect on the formation of large crystals of CPO‐27‐Ni and does not contribute to the framework composition.

Other studies on the synthesis of CPO‐27‐M have identified monomeric species as competing phases in the synthesis, proving that this framework is sensitive towards slight changes in the reaction conditions.[^49^, ^57^, ^58^] As we only observe the formation of a single phase, we propose that CPO‐27‐Ni is the thermodynamic product under these synthesis conditions and the presence of a competing linker promotes crystal growth through nucleation.

**Figure 2:**
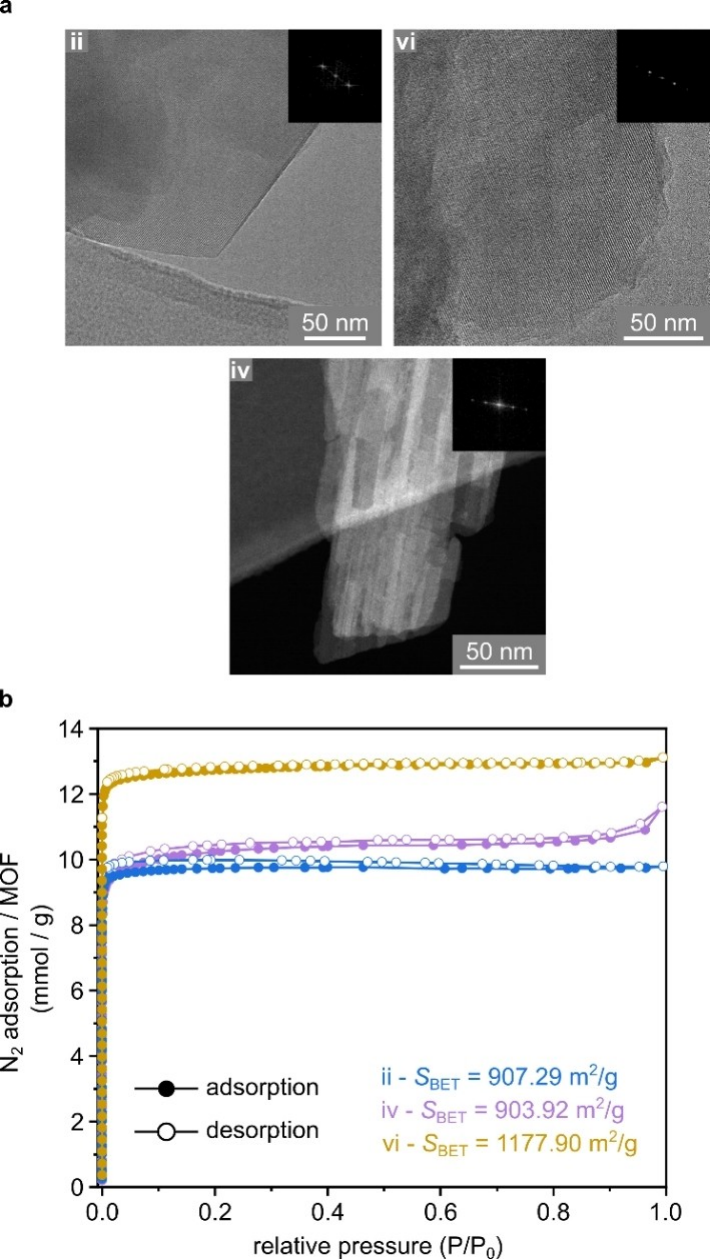
TEM images with FFT as inserts of CPO‐27‐Ni samples obtained via excess dhtp (**ii**), mixed dhtp/dhip (**vi**) synthesis, and STEM image with FFT insert of a Ni‐4,6‐dhip sample (**iv**). a) TEM images with FFT as inserts of CPO‐27‐Ni samples obtained via excess dhtp (**ii**), mixed dhtp/dhip (**vi**) synthesis, and STEM image with FFT insert of a Ni‐4,6‐dhip sample (**iv**)and b) N_2_‐isotherms and resulting BET areas of samples **ii**, **iv**, and **vi**.

Further comparative characterisation of the products **ii**, **iv**, and **vi** was performed using TEM and BET analyses. Figure 2a shows TEM images of crystals from syntheses with excess dhtp (Figure 2a‐**ii**), the structural analogue Ni‐4,6‐dhip (Figure 2a‐**iv**), and the dhtp/dhip‐mix approach (Figure 2a‐**vi**). All materials show high crystallinity throughout the analysed particles, with no indication of impurities. Figure 2b shows the respective nitrogen sorption data. All materials tested display type I isotherm behaviour,^[59]^ indicating microporous character.

BET surface areas of *S*_BET_=907.29 m^2^/g, 903.92 m^2^/g, and 1177.90 m^2^/g were recorded for **ii**, Ni‐4,6‐dhip **iv**, and **vi** respectively, which is relatively high, but still within the expected range for these materials. A total pore volume of 0.330 cm^3^/g, 0.326 cm^3^/g and 0.419 cm^3^/g was determined via the t‐plot method for **ii**, **iv**, and **vi**, respectively. This is in good agreement with previous observations^[36]^ and further supports the interpretations given above of a pure phase, highly crystalline product for **vi**. The d‐spacing calculated from TEM Fast Fourier Transformations (FFTs) were 1.31, 1.30, and 1.29 nm, for **ii**, **iv**, and **vi**, respectively. This corresponds with ($2\overset{-}{1}0$ ) reflection of a CPO‐27 framework with theoretical d‐spacing of 1.30 nm. TEM images (Figure 2a) show that the materials **ii** and **vi** have a needle‐like single‐crystal morphology (tens of microns long and 5–10 μm thick), while Ni‐4,6‐dhip (**iv**) crystallises as agglomerates of individual thin needles (10–30 nm thick). This agglomeration may also explain the small hysteresis gap seen for **iv** in the N_2_ isotherm at high relative pressures as individual crystals create interparticle space and therefore slow down the desorption. This is in good agreement with SEM images presented in Figure 1b.

### NO Storage and Release

**Figure 3:**
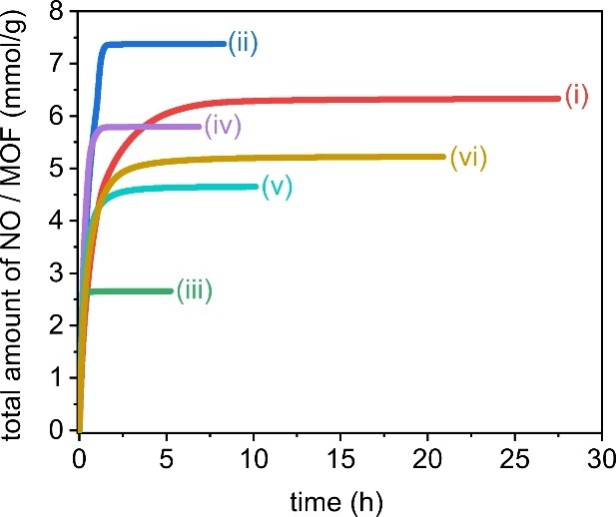
Nitric oxide release profiles of CPO‐27‐Ni materials of different crystallite sizes and the isostrucutral framework M‐4,6‐dhip (M=Co, Ni). **i**) CPO‐27‐Ni polycrystalline powder, **ii**) small single crystals of CPO‐27‐Ni, **iii**) medium sized single crystals CPO‐27‐Ni, **iv**) small single crystals of Ni‐4,6‐dhip, **v**) single crystals of Co‐4,6‐dhip, **vi**) large single crystals of CPO‐27‐Ni.

All syntheses yielded products that exhibit good crystallinity, hence open metal sites should be accessible upon dehydration. Previous studies have shown that an activation temperature of 150 °C is ideal for *in vacuo* removal of coordinated solvent to achieve a maximum NO uptake of ∼7 mmol/g in CPO‐27‐Ni powders.[^42^, ^43^] NO release profiles were recorded for the full range of synthesised materials, under a relative humidity of 11 %, and are displayed in Figure 3.

Water molecules diffuse through the crystallites, where NO is replaced at the metal centres and therefore a release of bound NO is triggered. The NO analysers record the NO release concentration in ppb/ppm. These values are then accumulated and integrated to achieve a total NO release amount (area under the curve), which is then normalised to the mass of MOF; NO release values are therefore expressed as mmol per gram of MOF and plotted against the duration of release. Highest release levels of NO are observed for **ii** and match the value for theoretical NO uptake; exact release values and durations are listed in Table S5. A slightly less efficient NO release was observed for **i** and **vi** showing longer release periods and lower NO values. The isostructural MOFs Ni‐4,6‐dhip (**iv**) and Co‐4,6‐dhip (**v**) both show high NO release levels. Sample **iii** shows lower NO release levels than all other materials and the shortest duration. The diffusion of water through the crystallites is essential for the release of bound NO. Small crystallites should promote faster diffusion and therefore display shorter release times. This trend is seen for samples **ii**, **iii**, **iv**, and **v**. Although, there is not a clear correlation between NO release duration and rising crystallite size, we do see a substantially longer NO release for the largest crystallite sample **vi**. Sample **i** also shows significant longer release times but this is attributed to agglomeration of the primary particles in the polycrystalline powder as seen in Figure 1b‐**i**. Despite the NO release differences, all materials display significant, high NO release levels that are suitable to trigger a biological response (pM–μM).[^22^, ^42^] While MOFs have been discussed as NO carriers for medical applications,[^22^, ^23^, ^60^] a mechanistic study of NO adsorption onto CPO‐27‐Ni framework has not yet been reported. In this study we performed *in situ* gas cell experiments on **vi** using synchrotron radiation.

### *In Situ* Gas Cell Experiments on CPO‐27‐Ni (vi)

During *in situ* gas cell experiments, only small structural differences are expected upon dehydration and NO‐adsorption of the MOF crystal, hence a powerful (bright and high resolution) X‐ray source is needed. To achieve sensible results, samples should display good diffraction and ideally be durable in the high energy beam. Single crystals obtained from the modulated mixed linker synthesis of **vi** yielded large and robust single crystals that meet all requirements for *in situ* gas cell experiments. A sacrificial crystal was used to develop an activation protocol for this system. Initially, the crystal was exposed to a strong vacuum (3.3×10^−6^ mbar) at room temperature (25 °C). The partial occupancy of the coordinated water molecule (Ni−O_w_) was tracked via SXRD to follow the process of dehydration. The water molecule resolved to full occupancy even after 1 h exposure to strong vacuum. The TGA analysis for that (bulk) sample (Figure S4‐**vi**) reveals an initial mass loss of approximately 31.8 % in the temperature range of 25–150 °C. Assuming a structure formula of [Ni_2_(C_8_H_2_O_6_) ⋅ (H_2_O)_2_] ⋅ 8H_2_O, this equates to the 8 loosely coordinated water molecules within the pores of the framework. The stronger bound water molecules (Ni−O_w_) of the partially dehydrated structure formula Ni_2_(C_8_H_2_O_6_) ⋅ (H_2_O)_2_ are only removed at higher temperatures, or in combination with applied vacuum.^[56]^ In order to test this hypothesis on a single crystal, the gas cell system was heated to 200 °C in increments. The activation proved to be much more susceptible towards elevated temperatures as 42 % of coordinated water was removed after just 45 min at 60 °C *in vacuo*. Although, this system proved to be extremely robust in the beam under those rather harsh conditions, the crystal decomposed at 200 °C *in vacuo*. A second crystal of **vi** was heated to 175 °C *in vacuo* (3.3×10^−6^ mbar) and a complete activation was achieved after just 4 h, as no residual electron density around the metal centre could be detected.

In the bulk phase of NO‐loaded CPO‐27‐Ni, bound NO is readily replaced by water as soon as the guest loaded MOF is introduced to moisture. In fact, as shown above (Figure 3), as little as 11 % relative humidity (RH) is enough to trigger such release. A preferable coordination of water over NO to the metal site can therefore be assumed. In order to avoid any re‐coordination of potentially remaining water molecules trapped in the pore system of the MOF or in the tubing of the gas cell, NO gas (2.5 bar absolute pressure) was introduced to the sample at high temperature (175 °C). The high temperature enhances movement through the lattice and maximises uptake, with the additional advantage of avoiding any residual water being re‐coordinated.

**Figure 4:**
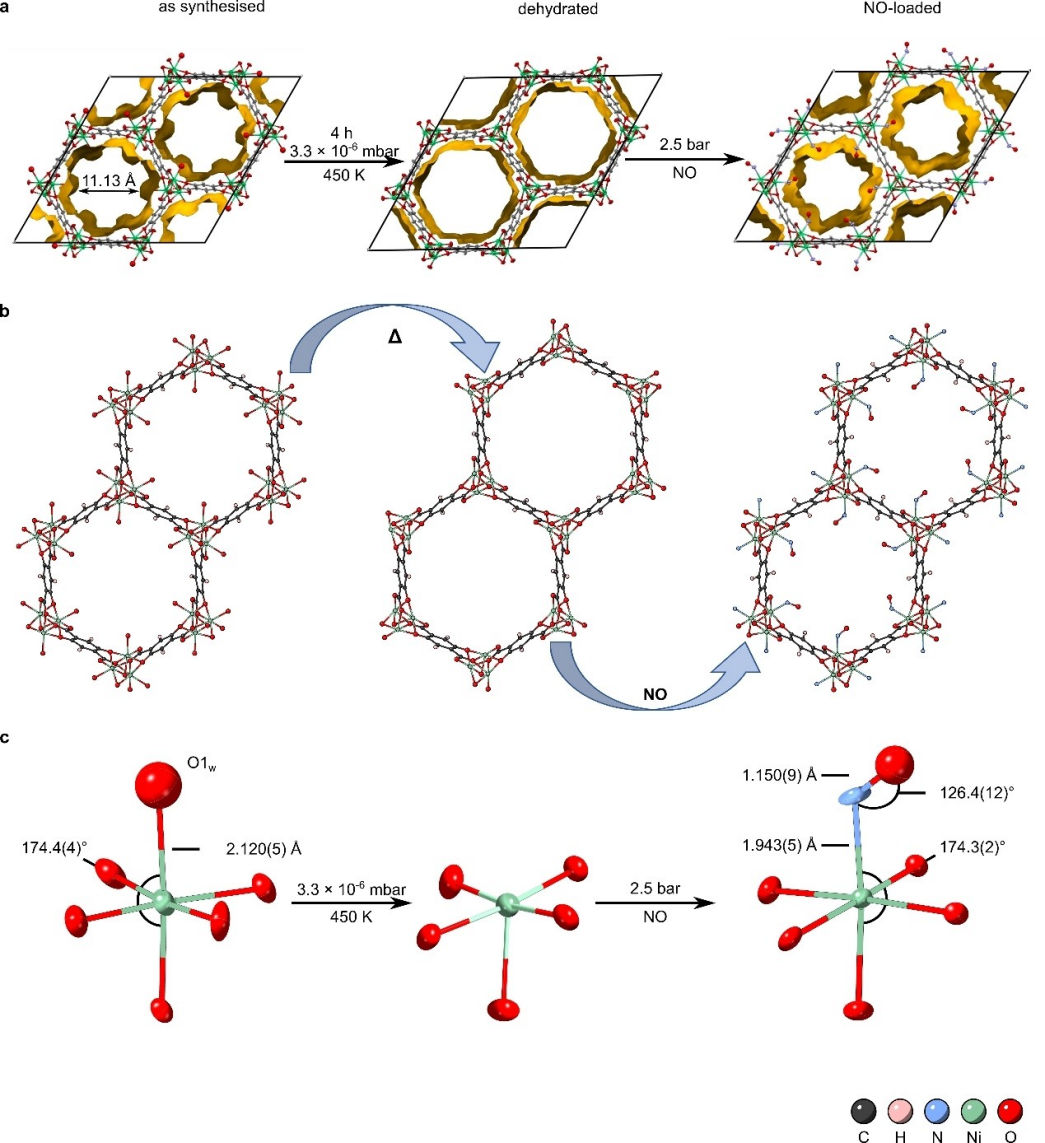
Activation process and nitric oxide loading on a single crystal of CPO‐27‐Ni (**vi**) viewed along the crystallographic *c*‐axis. Shown is the crystal structure model of the characteristic hexagonal pore channel of the as synthesised, dehydrated, and NO‐loaded structure with (a) calculated void space and (b) clarified picture of the dehydration process and (c) nickel oxygen cluster with important bond lengths and angles indicated. Data was acquired from a single‐crystal *in situ* gas cell experiment. Crystallographic data is available through the CCDC under no. 2060146, 2060150, and 2060151.

In theory, NO can occupy the CUSs in two binding motions, either in linear or bent conformation. In the former case a rather short bond length of ∼1.5 Å with high double bond character would be expected, while the bent conformation has a higher single bond character and therefore longer bond length. Long and co‐workers have studied the uptake of multiple guests on single crystals of CPO‐27‐Co. The guests O_2_ and CO_2_ both occupy the metal site in a bent formation and display bond lengths of 2.216(5) Å for O_2_ and 2.261(9) Å for CO_2_, respectively.^[46]^ Figure 4 visualises the single‐crystal structure model of **vi** in the process of dehydration and NO‐loading. Deposition numbers 2060146 (for **vi** – as synthesised), 2060150 (for **vi** – dehydrated), and 2060151–2060152 (for **vi** – NO loaded) contain the supplementary crystallographic data for this paper. These data are provided free of charge by the joint Cambridge Crystallographic Data Centre and Fachinformationszentrum Karlsruhe Access Structures service. Access Structures service www.ccdc.cam.ac.uk/structures (data available through the CCDC. The crystal structure models allowed for a precise characterisation of the pores in all three confirmations (as synthesised, dehydrated, and NO‐loaded). The pristine structure model (CCDC 2060146) has an accessible pore volume of 44.0 % per unit cell (Figure 4a left panel). Complete removal of the coordinated water (CCDC 2060150) enlarges the accessible pore volume to 59.1 % (Figure 4a middle panel). The NO‐loaded structure (CCDC 2060151) occupies slightly more space but leaves the large pore still intact with a pore volume of 42.5 % (Figure 4a right panel). The octahedral bond angles of the central nickel atom change slightly towards obtuse angles upon coordination of NO (full tabulation of all bond lengths and angles is given in the Supporting Information). Figure 4c compares the nickel coordination centre of the as synthesised, dehydrated and NO‐loaded structure model, where important bond lengths and angles are highlighted.

It is worth to mention that the nickel‐nitrogen (Ni−N1_NO_) bond length with 1.943(5) Å is shorter than the nickel‐oxygen (Ni−O_w_) with 2.120(5) Å by 0.177 Å and shorter than the bond length of other guests in bent conformation (discussed above). Furthermore, in a restraint‐free refinement, the nitrogen‐oxygen (N_1_−O_NO_) bond length was found to be 1.156(4) Å, with a bond angle of 126.2(9)°. This is slightly shorter than the proposed nitrogen‐oxygen bond length and angle of 1.43(3) Å and 140.4(16)° from Rietveld refinements.^[16]^ Although, it should be noted that any residual moisture, due to incomplete dehydration of the bulk material could have led to disorder or a dual occupancy of NO along with H_2_O around the metal centre, which would artificially elongate *or* shorten this bond. Other studies on a cobalt exchanged zeolite (LTA) have reported a transition metal (TM) NO bond of 2.23(6) Å and a N−O_NO_ bond of 1.47(11) Å.^[61]^ Although in the regime of a single bond, the rather short Ni−N_NO_ bond length confirms the strong interaction of NO with the nickel site.

### Disorder Modelling

**Figure 5:**
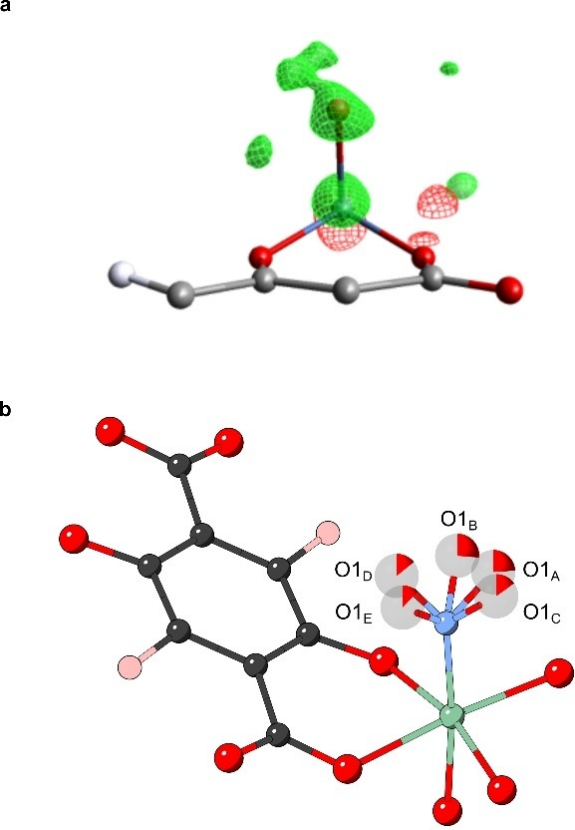
Visualisation of the CPO‐27‐Ni cluster. (a) Electron density difference Fourier Maps acquired from *in situ* single‐crystal gas cell experiments (dehydrated vs. NO‐loaded structure), additional electron density of unmodelled oxygen atoms of the nitrosyl group is indicated by the green wired spheres around the nickel metal site. Carbon atoms are displayed in light grey, hydrogen in white, oxygen in red, nickel in blue and the guest position in dark red. (b) Modelled disorder of the nitrosyl oxygen atoms of the NO‐loaded CPO‐27‐Ni structure. Nitrosyl oxygen atoms are disordered over five position (O1_A–E_) with varying partial occupancy factors, which are indicated as rendered pie chart. Carbon atoms are displayed in dark grey, hydrogen in rose, oxygen in red, nickel in green and nitrogen in blue. Crystallographic data is available through the CCDC under 2060151

As the Ni‐nitrosyl coordination is sensitive towards dual occupancy of potential water, an electron density difference (EDD) map was constructed between the dehydrated and the NO‐loaded structure model (NO group modelled as oxygen to indicate differences better). As the dehydrated structure has no electron density around the metal centre, differences should become clearly visible when compared to the NO‐loaded model. Figure 5a illustrates the obtained EDD map in wire view. Green areas represent electron density that has not yet been modelled while red areas indicate more density from modelled atoms than there is density in the data. In the case of a dual occupancy metal site (with both H_2_O and NO coordinated), the model would show additional unmodelled electron density in form of a distorted electron sphere around the coordinated guest molecule. As Figure 5a reveals, there is no additional unmodelled electron density around the guest other than a diffuse area that is the right distance for a potential O_NO_ atom. This also indicates that the O_NO_ is disordered over at least two positions around the central N_NO_. At first, the O_NO_ atoms were refined freely. After the assignment of the two disordered O_NO_ positions, additional electron density peaks became visible, which were assigned to further oxygen atoms of the same nitrosyl group. A total of five O_NO_ positions were identified each with an initial partial occupancy of 19.8 % (in a data set acquired at 25 °C). Disordered O_NO_ atoms were subsequently modelled in five different PARTs and since the combined occupancy of the O_NO_ atoms totalled 99 %, the SUMP (shelx) command was used, so the chemical occupancy of all five O_NO_ atoms was combined to 100 %. This also allowed for a free variable (occupancy) refinement for each individual position. Hence, favourable positions could be determined, and an accurate refinement of bond distances and angles was carried out. Figure 5b displays the NO structure model where all five O_NO_ positions are modelled and labelled as O1_A–E_. The partial occupancy of O1_A–E_ atoms is indicated as a rendered pie chart. It was found that O1_B_ (pointing into the pore channel along *c*‐axis) has the highest occupancy with 29.1(17)% and a Ni−N_NO_−O1_B_ bond angle of 125.6(10)° (all specific bond lengths and angles can be found in the crystallographic tables in the Supporting Information).

The EDD map analysis showed conclusively that only NO is bound at the metal centre and that the O_NO_ atoms are disordered around the N_NO_ atom over five positions. Although, moisture still trapped in the pores may be leading to a slight tilt of the thermal ellipsoid N_NO_. The N_NO_ ellipsoid was constrained using the RIGU parameter and N−O1_A–E_ bonds were optimised to 1.15 Å using the DFIX command.

### NO Desorption

**Figure 6:**
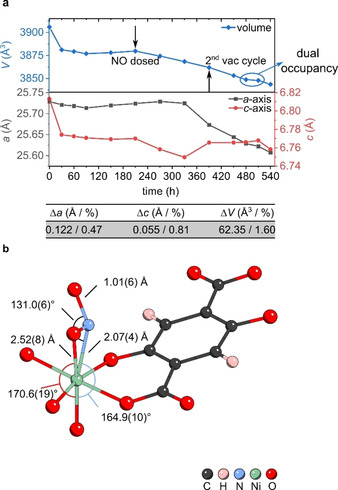
a) Change of lattice parameters of CPO‐27‐Ni (**vi**) followed on the same single crystal, when exposed to activating conditions and nitric oxide (NO). NO was dosed at 175 °C and the system was cooled down to room temperature for data collection. Subsequently the system was heated up again (175 °C) for desorption of NO (indicated as 2^nd^ vac cycle). b) visualisation of the Ni cluster in **vi** with a dual occupancy at the Ni site, where residual water is coordinated next to NO, with a partial occupancy of 7.1(12)% for water and 19.6(15)% for NO, respectively. Crystallographic data available through the CCDC under 2060152.

After successful NO‐loading, the same crystal was exposed to a second vacuum cycle in order to test for the desorption behaviour of NO. Following the process of dehydration (removal of water), NO adsorption (NO‐loading), and NO desorption (secondary activation), a slight shrinkage of the lattice parameters was observed. This has been previously investigated via spectroscopic methods and was described as a form of framework breathing upon dehydration.^[44]^ Figure 6a displays the variation of the CPO‐27‐Ni framework when exposed to activation and gas loading conditions, followed on the same single crystal. We confirm that the unit cell volume decreases slightly with longer exposure to strong vacuum and elevated temperatures and a total change of Δ*V*=1.6 % is observed. Furthermore, desorption of NO in a dry atmosphere proved to be harder than the removal of bound water molecules. After 3 h of activation, the N1_NO_ resolved to a partial occupancy of 19.6(15)%. Furthermore, the Ni1−N1_NO_ bond is elongated to 2.070(4) Å as opposed to 1.943(5) Å, while the N1−O1_NO_ shortened to 1.010(6) Å.

Despite the (large) error of the shortened N1−O1_NO_ bond, shortening of the discussed bond is indicative of NO slowly desorbing from the nickel centre as the bond lengths reflect more the character of uncoordinated NO gas. Interestingly, for this model a dual occupancy at the metal centre was observed as residual water was re‐coordinated with a partial occupancy of 7.1(12)% next to NO as visualised in Figure 6b. The Ni_1_−O1_w_ bond is significantly longer than in the as‐synthesised model with a bond length of 2.52(8) Å. This again indicates a rather weak coordination. As the system is left further under activation conditions, the partial occupancy of both NO and water is reduced and a complete removal of water is achieved. However, NO could not fully be removed after 4 h activation *in vacuo* at 175 °C and remained with a partial occupancy of 12.9(13)% for N1_NO_. This further confirms the strong interaction of the nickel metal centre in CPO‐27‐Ni with nitrogen containing guest molecules, such as NO. These strong interactions may be responsible for minor NO release inefficiencies (seen in Figure 3) but also makes this MOF ideal for the potential removal of other nitrogen containing molecules such as ammonia.^[27]^

## Conclusion

In this study we have reported a gradual increase of the crystallite size through a series of modulation in the synthesis of CPO‐27‐Ni. By offering the reaction mixture a further linker equivalent, modest increase in crystallite size (5 μm) was achieved. Interestingly, considerably larger single crystals were obtained for the first time via a novel mixed linker modulation approach, by using equimolar amounts of 2,5‐dhtp and the isomeric 4,6‐dhip ligand. PXRD, BET and TEM analysis proved high crystallinity and surface areas for this material. Furthermore, TGA and CHN analyses confirmed the desired molecular structure of [M_2_(ligand) ⋅ (H_2_O)_2_] ⋅ 8H_2_O. All techniques indicated phase purity (within their limitations), thus we propose that the competing 4,6‐dhip ligand only promotes single‐crystal growth of CPO‐27‐Ni (in **vi**) and is not incorporated into the framework. The full range of synthesised materials released high amounts (2.5–7 mmol/g) of NO in biologically active concentrations. Diffusion effects lead to a shorter NO release duration for smaller crystallites (<10 h), for the large crystallites, however, the release was significantly prolonged (21 h). A mechanistic study of NO adsorption onto large single crystals of CPO‐27‐Ni (**vi**) was investigated via *in situ* gas cell experiments. An efficient activation protocol was developed, yielding a dehydrated structure model after just 4 h at 175 °C *in vacuo*. For the first time, NO could be located in the single‐crystal structure of CPO‐27‐Ni. The Ni−N1_NO_ bond was found to be 1.943(5) Å, shorter than calculated via Rietveld methods for this system and as previously reported for a NO‐loaded Co‐exchanged zeolite LTA, but well within the range of NO‐loaded transition metal complexes.[^61^, ^62^] This further confirms the strong interaction of NO with the Ni metal centre in CPO‐27‐Ni. The N_NO_ is slightly distorted, which indicated a disorder of the NO‐group. After careful modelling of the disorder, five positions of O_NO_ were identified with varying partial occupancies. The preferred position (O1_B_) is pointing into the pore (along *c*‐direction) and resolved to a free occupancy factor of 29.1(17)%. Probing the subsequent desorption of NO indicated a slight shrinkage of the unit cell parameters. Furthermore, a dual occupancy at the Ni metal site was observed, where both NO and water were partially coordinated with free occupancy factor of 19.6(15)% for N1_NO_ and 7.1(12)% for O1_w_, respectively. However, elongated bond lengths for both gases indicated weaker coordination. Further exposure to activating conditions led to complete removal of water, but a full removal of NO could not be achieved in 4 h, which further proves the strong interaction of nitrogen containing guests with the nickel metal centre of CPO‐27. *In situ* gas cell experiments are an important tool to investigate guest‐host interactions in MOFs and crucial to fully understand applications that rely on these interactions. Single crystals of **vi** proved to be remarkably robust, well‐diffracting, and displayed an efficient activation, which makes this material especially promising for *in situ* single‐crystal studies of other (toxic) guests, such as ammonia.

## Conflict of interest

The authors declare no conflict of interest.

## Supporting information

As a service to our authors and readers, this journal provides supporting information supplied by the authors. Such materials are peer reviewed and may be re‐organized for online delivery, but are not copy‐edited or typeset. Technical support issues arising from supporting information (other than missing files) should be addressed to the authors.

Supplementary
