# Supplementary material for: Controlled Synthesis of Large Single Crystals of Metal‐Organic Framework CPO‐27‐Ni Prepared by a Modulation Approach: In situ Single‐Crystal X‐ray Diffraction Studies
Source: Chemistry. 2021 May 7;27(33):8537–46. doi: 10.1002/chem.202100528 (PMC8251849; doi:10.1002/chem.202100528)
Supplement: Supplementary file 1 — Supplementary [file CHEM-27-8537-s001.pdf]

Chemistry–A European Journal

Supporting Information

# Controlled Synthesis of Large Single Crystals of Metal-Organic Framework CPO-27-Ni Prepared by a Modulation Approach: *In situ* Single-Crystal X-ray Diffraction Studies

Simon M. Vornholt,* Caroline G. Elliott, Cameron M. Rice, Samantha E. Russell, Peter J. Kerr, Daniel N. Rainer, Michal Mazur, Mark R. Warren, Paul S. Wheatley, and Russell E. Morris

## Author Contributions

S.V. Formal analysis:Lead; Investigation:Lead; Project administration:Supporting; Writing – original draft:Lead; Writing – review & editing:Lead
C.E. Investigation:Supporting; Writing – review & editing:Supporting
C.R. Investigation:Supporting; Writing – review & editing:Supporting
S.R. Investigation:Supporting; Writing – review & editing:Supporting
P.K. Investigation:Supporting
D.R. Formal analysis:Supporting; Investigation:Supporting; Writing – review & editing:Supporting
M.M. Formal analysis:Supporting; Investigation:Supporting; Writing – review & editing:Supporting
M.W. Formal analysis:Supporting; Investigation:Supporting; Validation:Supporting; Writing – review & editing:Supporting
P.W. Data curation:Supporting; Project administration:Supporting; Validation:Supporting; Writing – review & editing:-Supporting
R.M. Project administration:Lead; Supervision:Lead; Validation:Lead; Writing – review & editing:Supporting

## Table of Contents

I. Experimental Procedures .......... 2
II. Results and Discussion .......... 3
III. Crystallographic Tables .......... 9
IV. Author Contributions .......... 21
V. References .......... 21

## I. Experimental Procedures

### Single Crystal X-ray Diffraction Experiments

*In situ* single crystal gas cell experiments were carried out on the I19 beamline at the Diamond Light Source, Rutherford Appleton Laboratory using synchrotron radiation (λ = 0.6889 Å). Crystals of **v** and **vi** were mounted on MiTiGen Microloops (50 µm) and secured with a non-diffracting two component epoxy glue (Loctite double bubble™). Care was taken to use as little glue as possible to ensure efficient gas transport through the crystal. The crystal mount with the secured crystal was inserted into a quartz capillary static cell with 1 mm outer diameter (set-up reported elsewhere).[1] The cell was then sealed appropriately, and the mount secured with aid of super glue in order to avoid movement during gas application. The cell was then connected to a gas control rig, with stainless-steel tubing. The dosage of gases was controlled via mass flow controllers. The crystals were dehydrated via a combination of heat and vacuum, using an Oxford Cryosystems cryostream at 175 °C, with a heating ramp of 360 °C/h, *in vacuo* ($3.5\times10^{-6}$ mbar) for 4 h. Datasets were recorded every 30 min to track the level of dehydration. Datasets were collected on a four circle Newport diffractometer equipped with a Pilatus 300K using synchrotron radiation (λ = 0.6889 Å). Each dataset consisted of three 124° omega scans at different phi orientation; each scan contained 620 images of 0.2° 2θ with 0.2 second exposure time. Once the dehydration was completed, dry nitric oxide was introduced at 2.5 bar absolute pressure. The system was then cooled down to 25 °C, with a dwell time of 30 min to allow for equilibration. Raw data were processed via Xia2[2] with aimless correction. Structures were solved using Intrinsic Phasing methods with shelXT[3] and refined with shelXL[4] in the Olex2 GUI.[5] Obtained crystal structures were visualised using either Mercury[6] or the CrystalMaker[7] software kit.

### Powder X-Ray Diffraction

Bulk samples were loaded into quartz glass capillaries (0.5 mm diameter) and measured on a STOE STADIP diffractometer, equipped with a primary beam monochromator ($CuK_{\alpha1}$) and a scintillation position-sensitive linear detector. Data were recorded at room temperature from 3-50° 2θ with a step size of 0.75 and a dwell time of 75 seconds in Debye-Scherrer mode.

### Thermogravimetric Analysis

Thermogravimetric analyses were carried out on a Stanton Redcroft STA-780 simultaneous TG-DTA in oxidising atmosphere (25 mL/min). Samples were heated to 700 °C at a heating ramp of 5 °C/min, with an isothermal step at 25 °C for 5 min.

### Composition Analysis (CHN)

Compositional analysis was carried out on a Thermo Fisher Scientific Flash SMART apparatus equipped with a stainless-steel CN/CHN separation column (2 m). Samples were analysed in a quartz reaction tube under oxygen (240 mL/min) at a furnace temperature of 950 °C, an oven temperature of 60 °C, and a run time of 600 s. Helium was applied as a carrier gas (140 mL/min).

### Scanning Electron Microscopy

Scanning electron microscopy micrographs were recorded on a FEI Scios Dualbeam, equipped with a Schottky FEG electron source. Samples were placed on an adhesive carbon tab, brushed with silver paste, and gold sputter coated using a Quorum Q150R ES system using 15 mA/30 s. The SEM was operated at 3 kV and 0.1 nA to ensure sensitive mapping of the surface. Images were recorded at 2500-3000× magnifications and a working distance of 5 mm.

**Transmission Electron Microscopy**

Transmission and scanning transmission electron microscopy were performed on a $C_s$-corrected FEI Titan Themis 200 TEM, equipped with a TVIPS 16 mega-pixel CMOS camera and a JEOL JEM NEOARM-200F TEM, equipped with a TVIPS CMOS XF416 camera. Both machines were operated at 200 kV. The samples were prepared by a conventional dropping method, using acetone as a dispersion medium and a holey-carbon coated copper TEM-grid.

**Nitrogen Adsorption Isotherms**

BET surface areas were determined from nitrogen adsorption isotherms, recorded on a Micromeritics ASAP 2020 Accelerated Surface Area and Porosimetry System, according to the Rouquerol theory.[8] Samples (100 mg) were heated to 150 °C *in vacuo* for 16 h with a heating rate of 5 °C/min.

**Activation of Bulk Samples and NO Loading and Release**

The NO storage and release capacities were assessed for each material. Samples (10–15 mg) were activated for 16 h (150 °C, $10^{-4}$ mbar) and subsequently bathed in a NO atmosphere (2 bar absolute pressure) for 1 h at room temperature. Physisorbed NO was removed by applying two vacuum/argon cycles. The samples were then assessed by a Sievers NOA 280i chemiluminescence analyser, as reported elsewhere.[9,10] Nitrogen (200 mL/min) with a relative humidity of 11%, was used as a carrier gas to expel bound nitric oxide at the metal centres. The raw data recorded in ppb/ppm was plotted in total amount of NO released per gram of MOF (mmol/g). Data were cut once the concentration dropped below a value of 20 ppb as this is near the detection limit of the analysers.

## II. Results and Discussion

All chemicals were purchased from common vendors and used without further purification (see Table S1).

**Table S1.** Tabulation of chemicals, with their respective vendors and CAS numbers, used for the synthesis of discussed frameworks and linkers.

| Chemical | CAS-number | Supplier |
|---|---|---|
| Hydroquinone synthesis, grade | 123-31-9 | Sigma-Aldrich |
| Resorcinol, 99.0% | 108-46-3 | Sigma-Aldrich |
| Potassium bicarbonate, 99.7% | 298-14-6 | Sigma-Aldrich |
| Hydrochloric acid, conc. | 7647-01-0 | Fisher Scientific |
| $CO_2$ (g) | 124-38-9 | Linde |
| Nickel acetate tetrahydrate, 99+% | 6018-89-9 | Acros Organics |
| Cobalt acetate tetrahydrate, 99+% | 6147-53-1 | Acros Organics |

| | | |
|---|---|---|
| Benzoic acid, 99.5% | 65-85-0 | Sigma-Aldrich |
| 2,5-dihydroxyterephthalic acid | 610-92-4 | Dragon Chemical Group Co. |

**Synthesis of 2,5-dihydroxyterephthalic acid (2,5-dhtp) & 4,6-dihydroxyisophthalic acid (4,6-dhip)**

The dihydroxycarboxylic acids were synthesised from their benzene diol precursors (hydroquinone for 2,5-dhtp and resorcinol for 4,6-dhip) following a simple Kolbe-Schmitt reaction, at 225 °C and 10 bar $CO_2$ in excess of $KHCO_3$, as reported elsewhere.[11,12] The reaction was carried out in an Asynt stainless-steel reactor, heated by a solid heating block. The obtained red/brown solid was dispersed in water (300 mL) and acidified with conc. HCl to reach a pH < 2. The beige precipitate was filtered and washed until the filtrate showed a pH > 5. The beige 4,6-dhip ligand (85% yield) was used without further purification; the yellow-brown 2,5-dhtp ligand (70% yield) was recrystallised from a 2:1 v:v $H_2O$:ethanol mixture.

**MOF Synthesis**

Commercially available 2,5-dhtp was used if not specified in the procedure. All materials were washed with water and ethanol (150 mL each). Table S2 shows an overview of all synthesis conditions used for materials i-vi.

(**i**) CPO-27-Ni, powder: In a Teflon liner (500 mL), nickel acetate tetrahydrate (13.1 mmol) was dissolved in $H_2O$ (175 mL). A solution of 2,5-dhtp (37.5 mmol) in tetrahydrofuran (THF) (175 mL) was then added and left to stir for 15 min. Subsequently, the autoclave was sealed and placed in the oven for 3 days at 110 °C; 91% yield based on nickel salt.

(**ii**) CPO-27-Ni, small single crystal rods (3–5 µm long): Obtained following the same procedure as MOF synthesis (**i**) with equimolar ratios of linker and metal salt and a reaction time of 5 days (110 °C); 33% yield based on nickel salt.

(**iii**) CPO-27-Ni, medium sized single crystals (20–30 µm): The in-house synthesised and recrystallised 2,5-dhtp linker was used in an equimolar solvothermal synthesis approach of CPO-27-Ni. The mixture was left to react at 130 °C for 4 days and a 33% yield based on nickel salt was achieved.

(**iv**) Ni-4,6-dhip, single crystal needles (10 µm): Nickel acetate tetrahydrate (1 mmol) was dissolved in water (10 mL) and added to a Teflon lined stainless steel autoclave (50 mL). A solution of in-house synthesised 4,6-dhip (1 mmol) in THF (10 mL) was added and the mixture was left to stir for 15 min. The liner was then capped and left to react for 4 days at 130 °C. Pale green single crystal needles with 70% yield (based on Ni) were obtained.

(**v**) Co-4,6-dhip, single crystal (30–50 µm): In a Teflon liner (30 mL), cobalt acetate tetrahydrate (1 mmol) and benzoic acid (4 mmol) were dissolved in a water/ethanol solvent mixture (5 mL each). The linker 4,6-dhip (1 mmol) was dissolved in THF (10 mL), slowly added to the salt/modulator mixture, and left to stir for 15 min. The Teflon liner was then capped and left to react for 3 days at 150 °C. Pink single crystals were obtained after filtration (70% yield based on Co).

(**vi**) CPO-27-Ni, large single crystals (50–100 µm): Synthesised following a linker modulation approach. Nickel acetate tetrahydrate (251 mg, 1 mmol) was dissolved in water (30 mL) and added to a Teflon liner (50 mL). 2,5-dihydroxyterephthalic acid (0.5 mmol) and 4,6-dihydroxyterephthalic acid (0.5 mmol) were added to the liner and left to stir for 15 min. The liner was then capped, sealed in an autoclave and placed in the oven for 3 days at 130 °C. Yellow-brown, rectangular rods of CPO-27-Ni, with a yield of 73% (based on nickel salt), were obtained after filtration.

**Table S2**. Overview of the synthesis conditions of the discussed frameworks **i**-**vi**.

| Sample ID / Conditions | **i** – CPO-27-Ni powder | **ii** – CPO-27-Ni small | **iii** – CPO-27-Ni medium | **iv** – Ni-4,6-dhip | **v** – Co-4,6-dhip | **vi** – CPO-27-Ni large |
|---|---|---|---|---|---|---|
| Metal salt (mmol) | 75 | 75 | 1 | 1 | 1 | 1 |
| 2,5-dhtp (mmol) | 37.5 | 75 | 1 | - | - | 0.5 |
| 4,6-dhip (mmol) | - | - | - | 1 | 1 | 0.5 |
| Benzoic acid (mmol) | - | - | - | - | 4 | - |

| | | | | | | |
|---|---|---|---|---|---|---|
| Ethanol (mL) | - | - | - | - | 5 | - |
| THF (mL) | 175 | 175 | 10 | 10 | 10 | - |
| Water (mL) | 175 | 175 | 10 | 10 | 5 | 30 |
| Reaction time (days) | 3 | 5 | 4 | 4 | 3 | 3 |
| Reaction temperature (°C) | 110 | 110 | 130 | 130 | 150 | 130 |

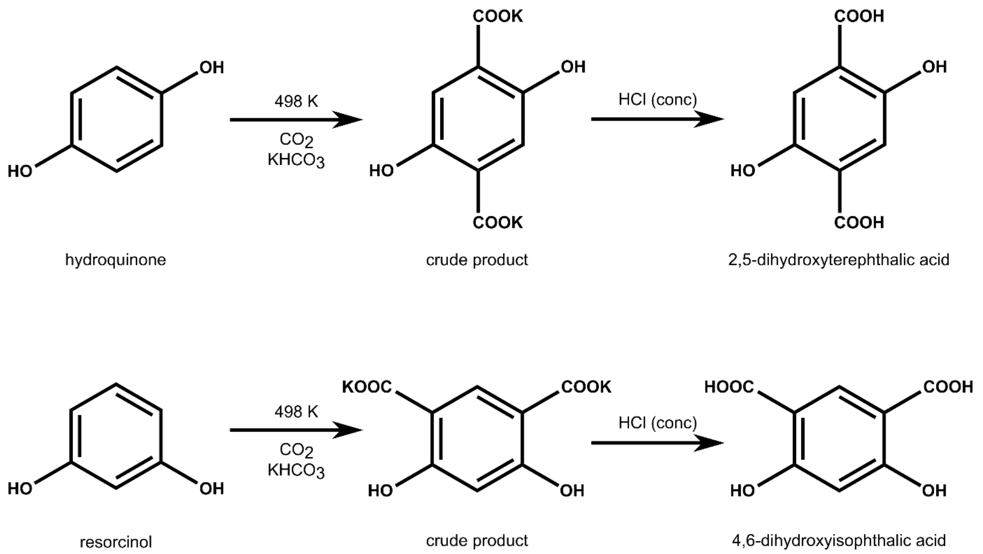


**Figure S1.** Synthesis scheme for linkers 2,5-dihydroxyterephthalic acid (2,5-dhtp) and 4,6-dihydroxyisophthalic acid (4,6-dhip) via a Kolbe-Schmitt reaction.

**Table S3.** Tabulation of LeBail refinements of CPO-27-Ni materials (**i**-**vi**). Materials **i**, **ii**, **iii**, and **vi** were compared to a unit cell of CPO-27-Co, while Ni-4,6-dhip (**iv**) was compared to a unit cell of Co-4,6-dhip (**v**) both acquired from single crystal diffraction (see Table S15).

| Sample | **unit cell parameters** | | | | **refinement quality** | | |
|---|---|---|---|---|---|---|---|
| | *a* (Å) | *b* (Å) | *c* (Å) | *V* (Å$^3$) | GooF | $R_p$ | $wR_p$ |
| CPO-27-Co reference (from CIF) | 26.0976(9) | 26.0976(9) | 6.7151(4) | 3960.8(4) | - | - | - |
| **i** – CPO-27-Ni stoichiometric | 25.9878(9) | 25.9878(9) | 6.6950(3) | 3915.8(2) | 5.17 | 6.37 | 9.04 |
| **ii** – CPO-27-Ni small sc | 25.9763(9) | 25.9763(9) | 6.6965(5) | 3911.6(2) | 4.36 | 5.9 | 7.76 |
| **iii** – CPO-27-Ni medium | 25.9475(9) | 25.9475(9) | 6.6836(3) | 3897.0(2) | 1.28 | 8.06 | 10.83 |
| **iv** – Ni-4,6-dhip medium sc | 25.8553(14) | 25.8553(14) | 6.7061(5) | 3882.4(5) | 2.88 | 10.39 | 17.13 |
| **vi** – CPO-27-Ni large sc | 25.9916(13) | 25.9916(13) | 6.6924(5) | 3915.(4) | 1.81 | 11.53 | 17.24 |
| **v** – Co-4,6-dhip reference (from CIF) | 26.1850(14) | 26.1850(14) | 6.7470(5) | 4006.3(5) | - | - | - |

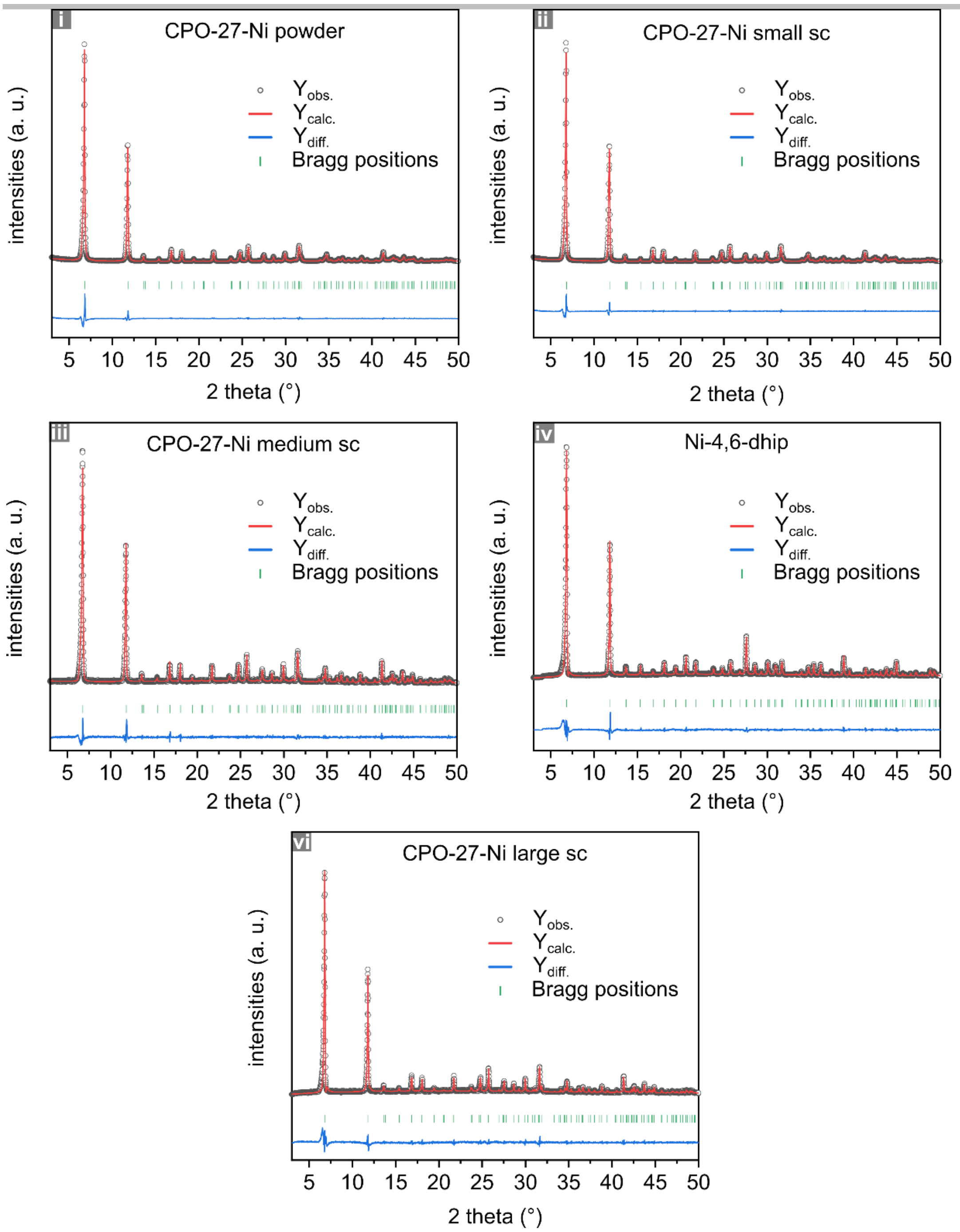


**Figure S2**. LeBail refinement plots of CPO-27-Ni materials (**i**-**vi**). Powder X-ray patterns ($Y_{observed}$ – hollow spheres) were acquired using Cu-radiation in Debye-Scherrer (transmission) mode.

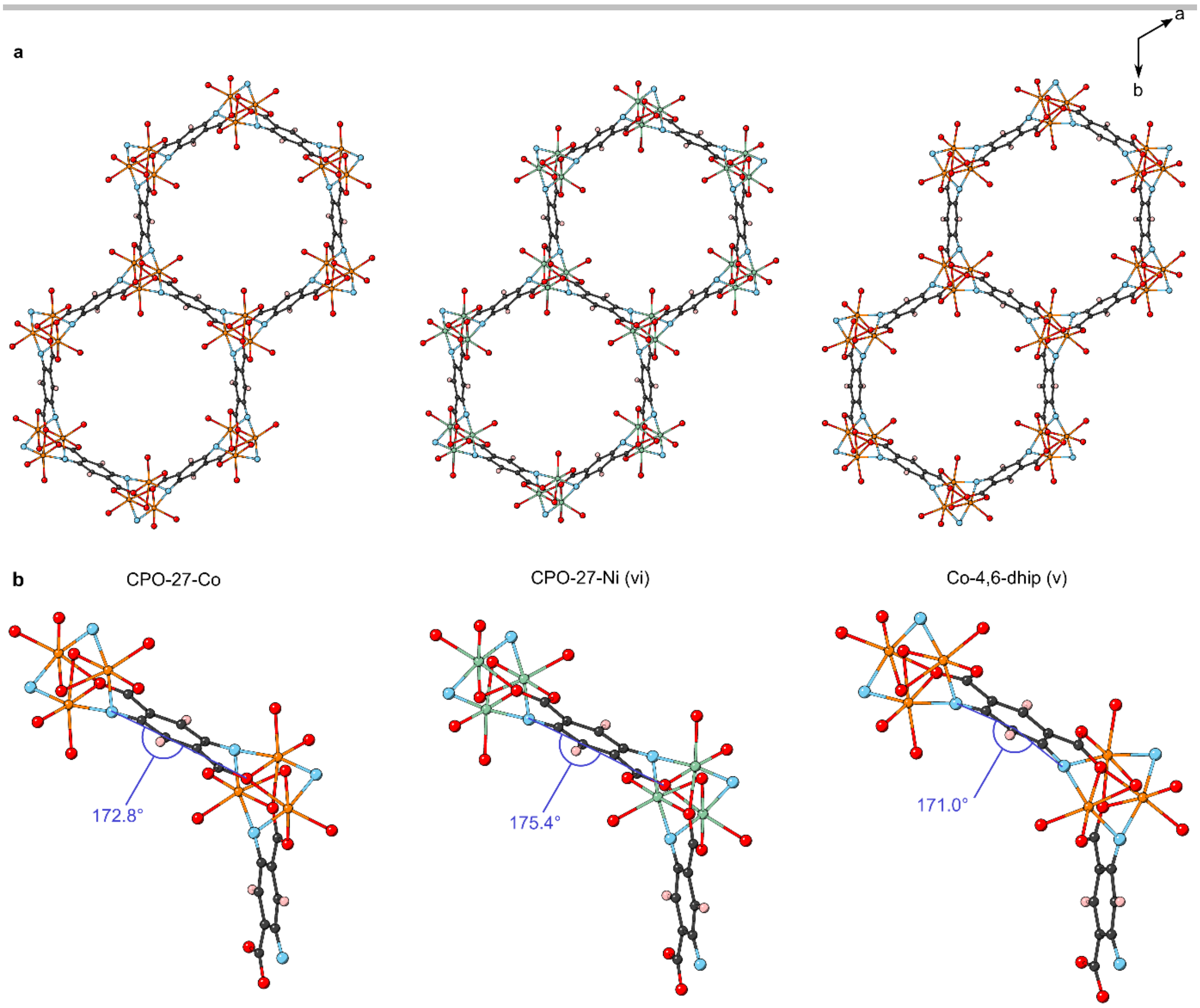


**Figure S3.** Comparison of the single crystal structure models of CPO-27-Co (left), mixed linker approach CPO-27-Ni-**vi** (middle), and Co-4,6-dhip (**v**) (right), with oxygen atoms of the hydroxy groups highlighted in light blue. Visualised is the hexagonal honeycomb pore (a) and a close-up of the linker coordination to the metal clusters (b), with resulting angles indicated in blue.

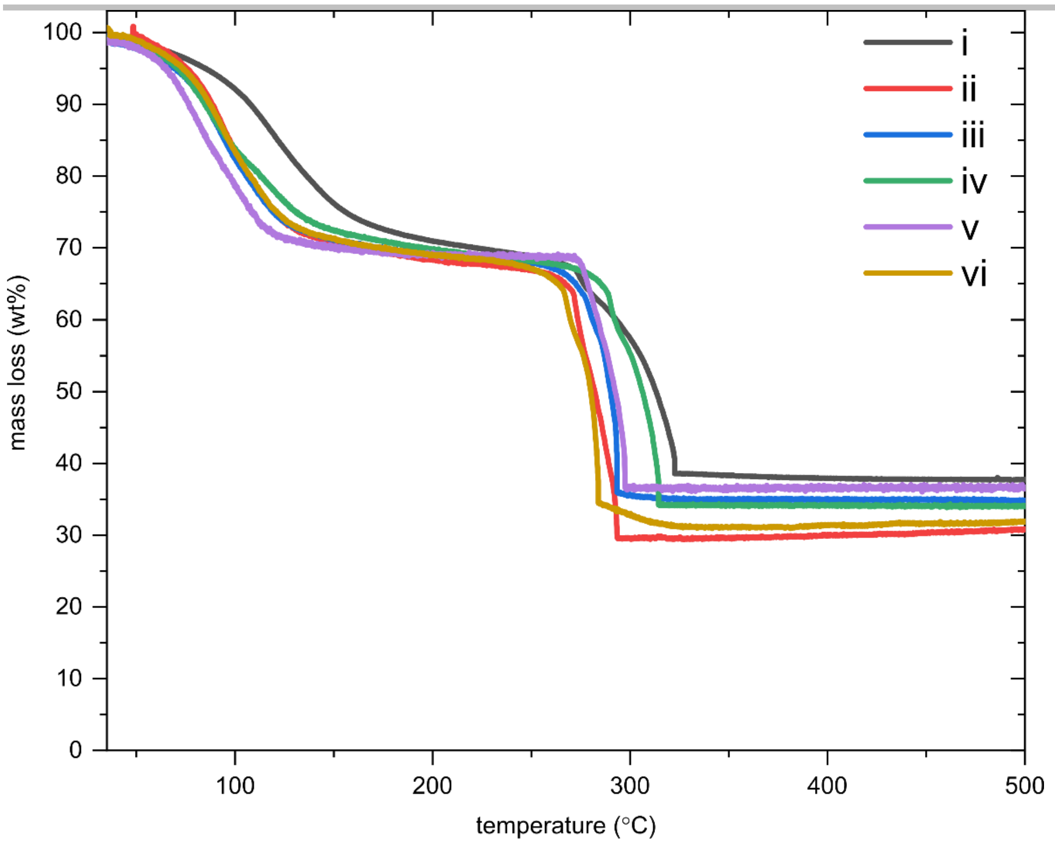


**Figure S4:** TGA profiles of discussed frameworks: **i** -**vi** in oxidising atmosphere and a heating ramp of 5 °C/min. With **i** – CPO-27-Ni (powder), **ii** – CPO-27-Ni (small), **iii** – CPO-27-Ni (medium), **iv** – Ni-4,6-dhip, **v** – Co-4,6-dhip, **vi** – CPO-27-Ni – large.

**Table S4. :** Tabulation of compositional analysis (CHN) of discussed frameworks with assumed MW of 491.63 g/mol ($Ni_2C_8H_{22}O_{16}$).

| Sample ID / Elemental Composition | $M_2C_8H_{22}O_{16}$ – expected | **i** – CPO-27-Ni powder | **ii** – CPO-27-Ni small | **iii** – CPO-27-Ni medium | **iv** – Ni-4,6-dhip | **v** – Co-4,6-dhip | **vi** – CPO-27-Ni large |
|---|---|---|---|---|---|---|---|
| Carbon Mean (%) | 19.54 | 20.42±0.51 | 19.89±0.18 | 20.70±0.08 | 19.83±0.17 | 20.81±0.03 | 20.77±0.08 |
| Hydrogen Mean (%) | 4.51 | 4.06±0.04 | 4.22±0.02 | 4.09±0.08 | 4.23±0.01 | 4.04±0.05 | 4.21±0.02 |
| Nitrogen Mean (%) | - | - | - | - | - | - | - |

**Table S5.** Total Nitric oxide (NO) release quantities and duration of release for the full range of synthesised materials. Measurements were stopped once the concentration fell below 20 ppb.

| Sample ID | Chemical Nature | Total NO released per gram of MOF (mmol / g) | Release Duration (h) |
|---|---|---|---|
| **i** | CPO-27-Ni - stoichiometric | 6.33 | 27.5 |
| **ii** | CPO-27-Ni - small single crystals (sc) | 7.35 | 8.2 |

| | | | | |
|---|---|---|---|---|
| **iii** | CPO-27-Ni medium sc | - | 2.66 | 5.2 |
| **iv** | Ni-4,6-dhip small sc | - | 5.8 | 7 |
| **v** | Co-4,6-dhip medium sc | - | 4.65 | 10 |
| **vi** | CPO-27-Ni large sc | - | 5.22 | 21 |

# III. Crystallographic Tables

The crystallographic data is available at the CCDC under the deposition numbers 2060146 (as synthesized), 2060150 (dehydrated), 2060151 (NO loaded), 2060152 (dual occupancy).

**Table S6.** Crystallographic table for CPO-27-Ni (**vi**) single crystal structure models acquired from *in situ* gas cell experiments.

| Identification code | CPO-27-Ni (**vi**) – as synthesised | CPO-27-Ni (**vi**) – dehydrated | CPO-27-Ni (**vi**) –NO loaded | CPO-27-Ni (**vi**) – dual occupancy |
|---|---|---|---|---|
| Empirical formula | $C_8H_6Ni_2O_8$ | $C_4HNiO_3$ | $C_4HNNiO_{3.98}$ | $C_4HN_{0.2}NiO_{3.12}$ |
| Formula weight | 347.55 | 155.76 | 185.51 | 160.17 |
| Temperature/K | 298.15 | 450 | 298.0 | 450 |
| Crystal system | trigonal | trigonal | trigonal | trigonal |
| Space group | R-3 | R-3 | R-3 | R-3 |
| a/Å | 25.7450(4) | 25.7018(6) | 25.7288(4) | 25.6288(7) |
| b/Å | 25.7450(4) | 25.7018(6) | 25.7288(4) | 25.6288(7) |
| c/Å | 6.80430(10) | 6.7719(2) | 6.75820(10) | 6.7660(2) |
| α/° | 90 | 90 | 90 | 90 |
| β/° | 90 | 90 | 90 | 90 |
| γ/° | 120 | 120 | 120 | 120 |
| Volume/$Å^3$ | 3905.71(13) | 3874.1(2) | 3874.36(13) | 3848.7(2) |
| Z | 9 | 18 | 18 | 18 |
| $\rho_{calc}g/cm^3$ | 1.330 | 1.202 | 1.431 | 1.244 |
| $\mu/mm^{-1}$ | 2.183 | 2.017 | 2.209 | 2.033 |
| F(000) | 1566.0 | 1386.0 | 1654.0 | 1426.0 |
| Crystal size/$mm^3$ | 0.05 × 0.02 × 0.01 | | | |
| Radiation | λ = 0.6889 | | | |
| 2Θ range for data collection/° | 3.066 to 61.574 | 6.096 to 61.648 | 6.106 to 61.69 | 3.08 to 61.426 |
| Index ranges | -38 ≤ h ≤ 37, -35 ≤ k ≤ 37, -10 ≤ l ≤ 10 | -27 ≤ h ≤ 37, -36 ≤ k ≤ 35, -10 ≤ l ≤ 9 | -25 ≤ h ≤ 36, -35 ≤ k ≤ 37, -9 ≤ l ≤ 10 | -33 ≤ h ≤ 29, -23 ≤ k ≤ 32, -7 ≤ l ≤ 10 |
| Reflections collected | 18198 | 8575 | 8586 | 6388 |
| Independent reflections | 2829 [$R_{int}$ = 0.0501, $R_{sigma}$ = 0.0476] | 2777 [$R_{int}$ = 0.0686, $R_{sigma}$ = 0.0974] | 2704 [$R_{int}$ = 0.0367, $R_{sigma}$ = 0.0421] | 2410 [$R_{int}$ = 0.0637, $R_{sigma}$ = 0.1090] |

| | | | | |
|---|---|---|---|---|
| Data/restraints/parameters | 2829/0/87 | 2777/0/74 | 2704/10/107 | 2410/0/85 |
| Goodness-of-fit on $F^2$ | 1.097 | 1.110 | 1.127 | 1.058 |
| Final R indexes [I>=2σ (I)] | $R_1$ = 0.0694, $wR_2$ = 0.2084 | $R_1$ = 0.0801, $wR_2$ = 0.2576 | $R_1$ = 0.0608, $wR_2$ = 0.1757 | $R_1$ = 0.0894, $wR_2$ = 0.2631 |
| Final R indexes [all data] | $R_1$ = 0.0827, $wR_2$ = 0.2145 | $R_1$ = 0.1018, $wR_2$ = 0.2647 | $R_1$ = 0.0677, $wR_2$ = 0.1777 | $R_1$ = 0.1567, $wR_2$ = 0.2878 |
| Largest diff. peak/hole/e $Å^{-3}$ | 2.90/-1.28 | 2.13/-1.40 | 1.83/-1.05 | 1.18/-0.72 |

**Table S7**. Specific bond lengths for the CPO-27-Ni (**vi**)_as synthesised single crystal structure model, acquired from *in situ* gas cell experiments.

| Atom | Atom | Length/Å | Atom | Atom | Length/Å |
|---|---|---|---|---|---|
| Ni1 | $Ni1^1$ | 2.9608(7) | O2 | C3 | 1.338(6) |
| Ni1 | $Ni1^2$ | 2.9610(7) | O3 | C1 | 1.266(6) |
| Ni1 | O2 | 1.995(4) | O4 | C1 | 1.253(5) |
| Ni1 | $O2^1$ | 2.037(4) | C1 | C2 | 1.493(6) |
| Ni1 | $O3^2$ | 2.054(3) | C3 | C4 | 1.398(7) |
| Ni1 | O3 | 2.019(3) | C3 | C2 | 1.428(6) |
| Ni1 | $O4^3$ | 2.030(4) | C4 | $C2^4$ | 1.407(7) |
| Ni1 | O1W | 2.120(5) | | | |

$^1$4/3-Y,2/3+X-Y,-1/3+Z; $^2$2/3+Y-X,4/3-X,1/3+Z; $^3$4/3-Y,2/3+X-Y,2/3+Z; $^4$1-X,1-Y,1-Z

**Table S8**. Specific bond angles for the CPO-27-Ni (**vi**)_as synthesised single crystal structure model, acquired from *in situ* gas cell experiments.

| **Atom** | **Atom** | **Atom** | **Angle/˚** | **Atom** | **Atom** | **Atom** | **Angle/˚** |
|---|---|---|---|---|---|---|---|
| $Ni1^1$ | Ni1 | $Ni1^2$ | 112.35(3) | $O4^3$ | Ni1 | $O2^1$ | 95.84(15) |
| $O2^1$ | Ni1 | $Ni1^1$ | 42.20(10) | $O4^3$ | Ni1 | $O3^2$ | 94.67(15) |
| O2 | Ni1 | $Ni1^2$ | 43.31(11) | $O4^3$ | Ni1 | O1W | 87.0(2) |
| $O2^1$ | Ni1 | $Ni1^2$ | 135.25(11) | O1W | Ni1 | $Ni1^2$ | 132.21(18) |
| O2 | Ni1 | $Ni1^1$ | 126.70(11) | O1W | Ni1 | $Ni1^1$ | 107.75(17) |
| O2 | Ni1 | $O2^1$ | 168.86(15) | Ni1 | O2 | $Ni1^2$ | 94.50(15) |

| | | | | | | | |
|---|---|---|---|---|---|---|---|
| O2 | Ni1 | O3[2] | 82.97(16) | C3 | O2 | Ni1[2] | 122.7(3) |
| O2[1] | Ni1 | O3[2] | 93.00(15) | C3 | O2 | Ni1 | 123.4(3) |
| O2 | Ni1 | O3 | 86.42(14) | Ni1 | O3 | Ni1[1] | 93.26(15) |
| O2 | Ni1 | O4[3] | 94.83(15) | C1 | O3 | Ni1 | 129.4(3) |
| O2 | Ni1 | O1W | 91.5(2) | C1 | O3 | Ni1[1] | 133.8(3) |
| O2[1] | Ni1 | O1W | 92.2(2) | C1 | O4 | Ni1[4] | 123.6(3) |
| O3[2] | Ni1 | Ni1[2] | 42.91(10) | O3 | C1 | C2 | 117.9(4) |
| O3[2] | Ni1 | Ni1[1] | 74.91(10) | O4 | C1 | O3 | 122.8(4) |
| O3 | Ni1 | Ni1[2] | 96.11(11) | O4 | C1 | C2 | 119.3(4) |
| O3 | Ni1 | Ni1[1] | 43.82(10) | O2 | C3 | C4 | 117.1(4) |
| O3 | Ni1 | O2[1] | 82.79(14) | O2 | C3 | C2 | 125.8(4) |
| O3 | Ni1 | O3[2] | 83.37(4) | C4 | C3 | C2 | 117.0(4) |
| O3 | Ni1 | O4[3] | 177.54(16) | C3 | C4 | C2[5] | 123.7(4) |
| O3[2] | Ni1 | O1W | 174.4(2) | C3 | C2 | C1 | 123.6(4) |
| O3 | Ni1 | O1W | 95.1(2) | C4[5] | C2 | C1 | 117.0(4) |
| O4[3] | Ni1 | Ni1[2] | 83.41(10) | C4[5] | C2 | C3 | 119.3(4) |
| O4[3] | Ni1 | Ni1[1] | 134.23(15) | | | | |

[1]4/3-Y,2/3+X-Y,-1/3+Z; [2]2/3+Y-X,4/3-X,1/3+Z; [3]4/3-Y,2/3+X-Y,2/3+Z; [4]2/3+Y-X,4/3-X,-2/3+Z; [5]1-X,1-Y,1-Z

**Table S9**. Specific bond lengths for the CPO-27-Ni (**vi**)_dehydrated single crystal structure model, acquired from *in situ* gas cell experiments.

| Atom | Atom | Length/Å | Atom | Atom | Length/Å |
|---|---|---|---|---|---|
| Ni1 | Ni1[1] | 2.8959(8) | O1 | C3 | 1.337(8) |
| Ni1 | Ni1[2] | 2.8957(8) | O2 | C1 | 1.284(7) |

| | | | | | |
|---|---|---|---|---|---|
| Ni1 | O1[2] | 2.019(4) | O3 | C1 | 1.227(6) |
| Ni1 | O1 | 1.989(4) | C1 | C2 | 1.505(8) |
| Ni1 | O2 | 1.994(5) | C4 | C3 | 1.389(8) |
| Ni1 | O2[1] | 2.007(4) | C4 | C2[4] | 1.408(8) |
| Ni1 | O3[3] | 2.000(4) | C3 | C2 | 1.405(7) |

[1]2/3+Y-X,4/3-X,1/3+Z; [2]4/3-Y,2/3+X-Y,-1/3+Z; [3]4/3-Y,2/3+X-Y,2/3+Z; [4]1-X,1-Y,1-Z

**Table S10**. Specific bond angles for the CPO-27-Ni (**vi**)_dehydrated single crystal structure model, acquired from *in situ* gas cell experiments.

| Atom | Atom | Atom | Angle/° | Atom | Atom | Atom | Angle/° |
|---|---|---|---|---|---|---|---|
| Ni1[1] | Ni1 | Ni1[2] | 114.30(3) | O3[3] | Ni1 | O1[1] | 96.31(18) |
| O1[1] | Ni1 | Ni1[1] | 43.33(13) | O3[3] | Ni1 | O2[2] | 100.5(2) |
| O1 | Ni1 | Ni1[2] | 44.15(12) | Ni1 | O1 | Ni1[2] | 92.53(18) |
| O1 | Ni1 | Ni1[1] | 125.79(14) | C3 | O1 | Ni1[2] | 124.9(4) |
| O1[1] | Ni1 | Ni1[2] | 138.46(14) | C3 | O1 | Ni1 | 124.4(3) |
| O1 | Ni1 | O1[1] | 168.30(19) | Ni1 | O2 | Ni1[1] | 92.74(19) |
| O1 | Ni1 | O2[2] | 82.1(2) | C1 | O2 | Ni1[1] | 132.8(4) |
| O1 | Ni1 | O2 | 86.64(17) | C1 | O2 | Ni1 | 134.1(4) |
| O1 | Ni1 | O3[3] | 95.36(18) | C1 | O3 | Ni1[4] | 122.8(4) |
| O2[2] | Ni1 | Ni1[2] | 43.45(13) | O2 | C1 | C2 | 116.0(5) |
| O2[2] | Ni1 | Ni1[1] | 74.66(13) | O3 | C1 | O2 | 122.0(5) |
| O2 | Ni1 | Ni1[2] | 101.40(16) | O3 | C1 | C2 | 122.0(5) |
| O2 | Ni1 | Ni1[1] | 43.81(13) | C3 | C4 | C2[5] | 122.9(5) |
| O2[2] | Ni1 | O1[1] | 96.61(19) | O1 | C3 | C4 | 116.4(5) |

| | | | | | | | |
|---|---|---|---|---|---|---|---|
| O2 | Ni1 | O1[1] | 81.67(18) | O1 | C3 | C2 | 127.0(5) |
| O2 | Ni1 | O2[2] | 85.14(7) | C4 | C3 | C2 | 116.6(6) |
| O2 | Ni1 | O3[3] | 174.2(2) | C4[5] | C2 | C1 | 115.6(5) |
| O3[3] | Ni1 | Ni1[2] | 83.76(12) | C3 | C2 | C1 | 123.9(5) |
| O3[3] | Ni1 | Ni1[1] | 136.35(15) | C3 | C2 | C4[5] | 120.4(5) |

[1]4/3-Y,2/3+X-Y,-1/3+Z; [2]2/3+Y-X,4/3-X,1/3+Z; [3]4/3-Y,2/3+X-Y,2/3+Z; [4]2/3+Y-X,4/3-X,-2/3+Z; [5]1-X,1-Y,1-Z

**Table S11**. Specific bond lengths for the CPO-27-Ni (**vi**)_NO loaded single crystal structure model, acquired from *in situ* gas cell experiments.

| Atom | Atom | Length/Å | Atom | Atom | Length/Å |
|---|---|---|---|---|---|
| Ni1 | Ni1[1] | 2.9871(6) | O4 | C1 | 1.250(5) |
| Ni1 | Ni1[2] | 2.9870(6) | C1 | C2 | 1.487(6) |
| Ni1 | O3[1] | 2.018(3) | C3 | C2 | 1.426(6) |
| Ni1 | O3 | 1.977(3) | C3 | C4 | 1.404(6) |
| Ni1 | O2 | 2.021(3) | C2 | C4[4] | 1.387(6) |
| Ni1 | O2[2] | 2.077(3) | N1 | O1A | 1.140(9) |
| Ni1 | O4[3] | 2.017(3) | N1 | O1B | 1.153(9) |
| Ni1 | N1 | 1.943(5) | N1 | O1C | 1.150(10) |
| O3 | C3 | 1.345(5) | N1 | O1D | 1.144(10) |
| O2 | C1 | 1.278(5) | N1 | O1E | 1.145(10) |

[1]2/3+Y-X,4/3-X,1/3+Z; [2]4/3-Y,2/3+X-Y,-1/3+Z; [3]2/3+Y-X,4/3-X,-2/3+Z; [4]4/3-X,5/3-Y,2/3-Z

**Table S12.** Specific bond angles for the CPO-27-Ni (**vi**)_NO loaded single crystal structure model, acquired from *in situ* gas cell experiments.

| Atom | Atom | Atom | Angle/° | Atom | Atom | Atom | Angle/° |
|---|---|---|---|---|---|---|---|
| Ni1[1] | Ni1 | Ni1[2] | 110.68(2) | N1 | Ni1 | O2[1] | 174.3(2) |

| | | | | | | | |
|---|---|---|---|---|---|---|---|
| $O3^{2}$ | Ni1 | $Ni1^{2}$ | 41.09(9) | N1 | Ni1 | O2 | 95.45(19) |
| $O3^{2}$ | Ni1 | $Ni1^{1}$ | 133.29(10) | N1 | Ni1 | $O4^{3}$ | 89.11(19) |
| O3 | Ni1 | $Ni1^{2}$ | 126.38(10) | Ni1 | O3 | $Ni1^{1}$ | 96.78(13) |
| O3 | Ni1 | $Ni1^{1}$ | 42.13(9) | C3 | O3 | $Ni1^{1}$ | 124.3(3) |
| O3 | Ni1 | $O3^{2}$ | 167.39(12) | C3 | O3 | Ni1 | 121.9(3) |
| O3 | Ni1 | O2 | 86.41(13) | Ni1 | O2 | $Ni1^{2}$ | 93.57(13) |
| O3 | Ni1 | $O2^{1}$ | 81.80(13) | C1 | O2 | Ni1 | 127.5(3) |
| $O3^{2}$ | Ni1 | O2 | 82.21(13) | C1 | O2 | $Ni1^{2}$ | 134.6(3) |
| $O3^{2}$ | Ni1 | $O2^{1}$ | 91.23(13) | C1 | O4 | $Ni1^{4}$ | 123.9(3) |
| O3 | Ni1 | $O4^{3}$ | 95.53(13) | O2 | C1 | C2 | 118.4(4) |
| $O2^{1}$ | Ni1 | $Ni1^{1}$ | 42.49(9) | O4 | C1 | O2 | 122.3(4) |
| O2 | Ni1 | $Ni1^{2}$ | 43.95(9) | O4 | C1 | C2 | 119.3(4) |
| $O2^{1}$ | Ni1 | $Ni1^{2}$ | 73.77(9) | O3 | C3 | C2 | 125.7(4) |
| O2 | Ni1 | $Ni1^{1}$ | 94.21(10) | O3 | C3 | C4 | 117.0(4) |
| O2 | Ni1 | $O2^{1}$ | 81.95(4) | C4 | C3 | C2 | 117.4(4) |
| $O4^{3}$ | Ni1 | $Ni1^{2}$ | 132.35(11) | C3 | C2 | C1 | 123.3(4) |
| $O4^{3}$ | Ni1 | $Ni1^{1}$ | 84.19(9) | $C4^{5}$ | C2 | C1 | 117.3(4) |
| $O4^{3}$ | Ni1 | $O3^{2}$ | 95.39(13) | $C4^{5}$ | C2 | C3 | 119.3(4) |
| $O4^{3}$ | Ni1 | $O2^{1}$ | 93.70(13) | $C2^{5}$ | C4 | C3 | 123.3(4) |
| $O4^{3}$ | Ni1 | O2 | 174.96(14) | O1A | N1 | Ni1 | 129.2(12) |
| N1 | Ni1 | $Ni1^{2}$ | 107.85(16) | O1B | N1 | Ni1 | 125.8(10) |
| N1 | Ni1 | $Ni1^{1}$ | 133.17(17) | O1C | N1 | Ni1 | 129.0(15) |
| N1 | Ni1 | $O3^{2}$ | 93.44(19) | O1D | N1 | Ni1 | 134.9(16) |

| | | | | | | | |
|---|---|---|---|---|---|---|---|
| N1 | Ni1 | O3 | 93.0(2) | O1E | N1 | Ni1 | 132.3(17) |

[1]4/3-Y,2/3+X-Y,-1/3+Z; [2]2/3+Y-X,4/3-X,1/3+Z; [3]2/3+Y-X,4/3-X,-2/3+Z; [4]4/3-Y,2/3+X-Y,2/3+Z; [5]4/3-X,5/3-Y,2/3-Z

**Table S13**. Specific bond lengths for the CPO-27-Ni (**vi**)_dual occupancy single crystal structure model, acquired from *in situ* gas cell experiments.

| Atom | Atom | Length/Å | Atom | Atom | Length/Å |
|---|---|---|---|---|---|
| Ni1 | Ni1[1] | 2.9109(10) | O2 | C2 | 1.349(9) |
| Ni1 | Ni1[2] | 2.9110(10) | O3 | C4 | 1.283(8) |
| Ni1 | O2 | 1.980(5) | O4 | C4 | 1.249(7) |
| Ni1 | O2[1] | 2.005(5) | C4 | C3 | 1.466(10) |
| Ni1 | O3[2] | 2.020(5) | C3 | C1[4] | 1.429(11) |
| Ni1 | O3 | 1.995(5) | C3 | C2 | 1.400(8) |
| Ni1 | O4[3] | 1.993(5) | C1 | C2 | 1.376(10) |
| Ni1 | O1W | 2.52(8) | N1 | O1NO | 1.01(6) |

[1]2/3+Y-X,4/3-X,1/3+Z; [2]4/3-Y,2/3+X-Y,-1/3+Z; [3]2/3+Y-X,4/3-X,-2/3+Z; [4]1-X,1-Y,1-Z

**Table S14**. Specific bond angles for the CPO-27-Ni (**vi**)_dual occupancy single crystal structure model, acquired from *in situ* gas cell experiments.

| Atom | Atom | Atom | Angle/° | Atom | Atom | Atom | Angle/° |
|---|---|---|---|---|---|---|---|
| Ni1[1] | Ni1 | Ni1[2] | 113.61(4) | O4[3] | Ni1 | O2[1] | 96.2(2) |
| O2 | Ni1 | Ni1[2] | 43.40(14) | O4[3] | Ni1 | O3[2] | 99.2(2) |
| O2[1] | Ni1 | Ni1[2] | 137.36(16) | O4[3] | Ni1 | O3 | 175.9(2) |
| O2 | Ni1 | Ni1[1] | 126.03(15) | O4[3] | Ni1 | O1W | 83.2(18) |
| O2[1] | Ni1 | Ni1[1] | 42.75(14) | O4[3] | Ni1 | N1 | 88.1(10) |

| | | | | | | | |
|---|---|---|---|---|---|---|---|
| O2 | Ni1 | O2[1] | 168.4(2) | O1W | Ni1 | Ni1[1] | 97.4(18) |
| O2 | Ni1 | O3 | 86.85(19) | N1 | Ni1 | Ni1[2] | 125.7(10) |
| O2[1] | Ni1 | O3[2] | 95.5(2) | N1 | Ni1 | Ni1[1] | 109.3(10) |
| O2 | Ni1 | O3[2] | 81.7(2) | Ni1 | O2 | Ni1[2] | 93.9(2) |
| O2 | Ni1 | O4[3] | 95.3(2) | C2 | O2 | Ni1 | 123.7(4) |
| O2 | Ni1 | O1W | 107.2(19) | C2 | O2 | Ni1[2] | 124.5(5) |
| O2[1] | Ni1 | O1W | 75.2(18) | Ni1 | O3 | Ni1[1] | 93.0(2) |
| O2 | Ni1 | N1 | 84.5(10) | C4 | O3 | Ni1[1] | 134.0(4) |
| O2[1] | Ni1 | N1 | 96.8(10) | C4 | O3 | Ni1 | 132.4(4) |
| O3 | Ni1 | Ni1[2] | 100.11(17) | C4 | O4 | Ni1[4] | 124.1(5) |
| O3 | Ni1 | Ni1[1] | 43.86(15) | O3 | C4 | C3 | 116.7(5) |
| O3[2] | Ni1 | Ni1[2] | 43.18(14) | O4 | C4 | O3 | 120.2(6) |
| O3[2] | Ni1 | Ni1[1] | 74.50(14) | O4 | C4 | C3 | 123.1(7) |
| O3 | Ni1 | O2[1] | 81.7(2) | C1[5] | C3 | C4 | 115.7(6) |
| O3 | Ni1 | O3[2] | 84.58(7) | C2 | C3 | C4 | 125.5(7) |
| O3 | Ni1 | O1W | 92.9(18) | C2 | C3 | C1[5] | 118.7(6) |
| O3[2] | Ni1 | O1W | 170.6(19) | C2 | C1 | C3[5] | 122.9(6) |
| O3[2] | Ni1 | N1 | 164.9(10) | O2 | C2 | C3 | 125.6(6) |
| O3 | Ni1 | N1 | 88.6(10) | O2 | C2 | C1 | 116.0(6) |
| O4[3] | Ni1 | Ni1[2] | 83.81(13) | C1 | C2 | C3 | 118.3(7) |
| O4[3] | Ni1 | Ni1[1] | 135.52(17) | O1NO | N1 | Ni1 | 132(4) |

[1]2/3+Y-X,4/3-X,1/3+Z; [2]4/3-Y,2/3+X-Y,-1/3+Z; [3]2/3+Y-X,4/3-X,-2/3+Z; [4]4/3-Y,2/3+X-Y,2/3+Z; [5]1-X,1-Y,1-Z

## Reference Structures

**Table S15**. Crystallographic tables of single crystal reference structure models used for comparison with CPO-27-Ni (**vi**).

| Identification code | CPO-27-Co | Co-4,6-dhip (v) |
|---|---|---|
| Empirical formula | $C_{4.24}H_{3.18}Co_{1.06}O_{5.96}$ | $C_4H_{2.39}CoO_{6.83}$ |
| Formula weight | 211.81 | 218.65 |
| Temperature/K | 293(2) | 293(2) |
| Crystal system | trigonal | trigonal |
| Space group | R-3 | R3m |
| a/Å | 26.0976(9) | 26.1850(14) |
| b/Å | 26.0976(9) | 26.1850(14) |
| c/Å | 6.7151(4) | 6.7470(5) |
| α/° | 90 | 90 |
| β/° | 90 | 90 |
| γ/° | 120 | 120 |
| Volume/Å$^3$ | 3960.8(4) | 4006.3(5) |
| Z | 16.99992 | 18 |
| $\rho_{calc}$g/cm$^3$ | 1.510 | 1.631 |
| μ/mm$^{-1}$ | 1.780 | 1.776 |
| F(000) | 1782.0 | 1944.0 |
| Crystal size/mm$^3$ | 0.04 × 0.01 × 0.01 | 0.05 × 0.02 × 0.01 |
| Radiation | λ = 0.6889 | λ = 0.6889 |
| 2Θ range for data collection/° | 3.026 to 62.546 | 5.224 to 60.884 |
| Index ranges | -38 ≤ h ≤ 39, -37 ≤ k ≤ 38, -9 ≤ l ≤ 6 | -30 ≤ h ≤ 37, -38 ≤ k ≤ 28, -9 ≤ l ≤ 9 |

| | | |
|---|---|---|
| Reflections collected | 14912 | 8679 |
| Independent reflections | 2889 [$R_{int}$ = 0.1072, $R_{sigma}$ = 0.0790] | 2896 [$R_{int}$ = 0.0423, $R_{sigma}$ = 0.0540] |
| Data/restraints/parameters | 2889/0/103 | 2896/1/140 |
| Goodness-of-fit on $F^2$ | 1.060 | 1.102 |
| Final R indexes [I>=2σ (I)] | $R_1$ = 0.0686, $wR_2$ = 0.1785 | $R_1$ = 0.0372, $wR_2$ = 0.0921 |
| Final R indexes [all data] | $R_1$ = 0.0971, $wR_2$ = 0.1966 | $R_1$ = 0.0398, $wR_2$ = 0.0930 |
| Largest diff. peak/hole / e Å$^{-3}$ | 1.72/-1.16 | 2.38/-0.79 |

**Table S16**. Specific bond lengths for the CPO-27-Co_reference structure single crystal structure model.

| Atom | Atom | Length/Å | Atom | Atom | Length/Å |
|---|---|---|---|---|---|
| Co1 | O2$^{1}$ | 2.037(2) | O4 | C4 | 1.273(4) |
| Co1 | O2 | 2.042(3) | O3 | C4 | 1.257(4) |
| Co1 | O4$^{2}$ | 2.059(3) | C4 | C3 | 1.490(5) |
| Co1 | O4$^{3}$ | 2.190(3) | C1 | C3$^{5}$ | 1.405(5) |
| Co1 | O3$^{4}$ | 2.044(3) | C1 | C2 | 1.399(5) |
| Co1 | O1W | 2.147(3) | C3 | C2 | 1.405(5) |
| O2 | C1 | 1.358(4) | | | |

$^{1}$1/3+Y-X,5/3-X,-1/3+Z; $^{2}$2/3-Y+X,1/3+X,4/3-Z; $^{3}$+Y,1-X+Y,1-Z; $^{4}$4/3-X,5/3-Y,2/3-Z; $^{5}$4/3-X,5/3-Y,5/3-Z

**Table S17**. Specific bond angles for the CPO-27-Co_reference structure single crystal structure model.

| Atom | Atom | Atom | Angle/˚ | Atom | Atom | Atom | Angle/˚ |
|---|---|---|---|---|---|---|---|
| O2$^{1}$ | Co1 | O2 | 166.68(9) | C1 | O2 | Co1 | 121.6(2) |
| O2$^{1}$ | Co1 | O4$^{2}$ | 81.62(10) | C1 | O2 | Co1$^{5}$ | 122.6(2) |
| O2$^{1}$ | Co1 | O4$^{3}$ | 84.34(10) | Co1$^{6}$ | O4 | Co1$^{7}$ | 91.79(9) |

| | | | | | | | |
|---|---|---|---|---|---|---|---|
| O2 | Co1 | O4[3] | 84.78(10) | C4 | O4 | Co1[7] | 136.0(2) |
| O2 | Co1 | O4[2] | 88.92(10) | C4 | O4 | Co1[6] | 128.1(2) |
| O2 | Co1 | O3[4] | 95.26(11) | C4 | O3 | Co1[4] | 123.0(2) |
| O2[1] | Co1 | O3[4] | 94.92(11) | O4 | C4 | C3 | 118.5(3) |
| O2[1] | Co1 | O1W | 92.85(13) | O3 | C4 | O4 | 122.0(3) |
| O2 | Co1 | O1W | 95.29(12) | O3 | C4 | C3 | 119.4(3) |
| O4[3] | Co1 | O4[2] | 79.81(3) | O2 | C1 | C3[8] | 125.0(3) |
| O4[3] | Co1 | O1W | 92.60(13) | O2 | C1 | C2 | 117.3(3) |
| O3[4] | Co1 | O4[3] | 175.07(11) | C2 | C1 | C3[8] | 117.7(3) |
| O3[4] | Co1 | O4[2] | 95.26(11) | C1[8] | C3 | C4 | 124.5(3) |
| O3[4] | Co1 | O1W | 92.31(13) | C2 | C3 | C4 | 116.6(3) |
| O1W | Co1 | O4[2] | 170.98(13) | C2 | C3 | C1[8] | 119.0(3) |
| Co1[5] | O2 | Co1 | 96.85(10) | C1 | C2 | C3 | 123.3(3) |

[1]1/3+Y-X,5/3-X,-1/3+Z; [2]+Y,1-X+Y,1-Z; [3]2/3-Y+X,1/3+X,4/3-Z; [4]4/3-X,5/3-Y,2/3-Z; [5]5/3-Y,4/3+X-Y,1/3+Z; [6]-1/3+Y,1/3-X+Y,4/3-Z; [7]1-Y+X,+X,1-Z; [8]4/3-X,5/3-Y,5/3-Z

**Table S18**. Specific bond lengths for the Co-4,6-dhip (**v**) structure single crystal structure model.

| Atom | Atom | Length/Å | Atom | Atom | Length/Å |
|---|---|---|---|---|---|
| Co1 | O4[1] | 2.082(3) | O3 | C1 | 1.281(5) |
| Co1 | O4[2] | 2.093(3) | C1 | C2 | 1.488(5) |
| Co1 | O2 | 2.018(3) | C5 | C4[4] | 1.405(4) |
| Co1 | O3[2] | 2.062(3) | C5 | C4 | 1.405(4) |
| Co1 | O3[3] | 2.165(3) | C3 | C2 | 1.390(5) |
| Co1 | O1W | 2.143(3) | C3 | C2[4] | 1.390(4) |

| | | | | | |
|---|---|---|---|---|---|
| O4 | C4 | 1.346(5) | C4 | C2 | 1.436(5) |
| O2 | C1 | 1.273(4) | | | |

[1]+X,+Y,1+Z; [2]4/3-Y,2/3+X-Y,2/3+Z; [3]2/3+Y-X,4/3-X,1/3+Z; [4]+X,1+X-Y,+Z

**Table S19**. Specific bond angles for the Co-4,6-dhip (**v**) structure single crystal structure model.

| Atom | Atom | Atom | Angle/° | Atom | Atom | Atom | Angle/° |
|---|---|---|---|---|---|---|---|
| O4$^{1}$ | Co1 | O4$^{2}$ | 168.27(11) | C4 | O4 | Co1$^{4}$ | 129.9(3) |
| O4$^{2}$ | Co1 | O3$^{3}$ | 83.01(12) | C1 | O2 | Co1 | 124.3(3) |
| O4$^{1}$ | Co1 | O3$^{3}$ | 89.43(12) | Co1$^{5}$ | O3 | Co1$^{6}$ | 93.10(12) |
| O4$^{1}$ | Co1 | O1W | 92.33(14) | C1 | O3 | Co1$^{6}$ | 133.2(2) |
| O4$^{2}$ | Co1 | O1W | 94.78(14) | C1 | O3 | Co1$^{5}$ | 125.0(3) |
| O2 | Co1 | O4$^{2}$ | 92.15(13) | O2 | C1 | O3 | 122.2(3) |
| O2 | Co1 | O4$^{1}$ | 97.12(13) | O2 | C1 | C2 | 118.2(4) |
| O2 | Co1 | O3$^{2}$ | 171.15(12) | O3 | C1 | C2 | 119.7(3) |
| O2 | Co1 | O3$^{3}$ | 92.28(12) | C4$^{7}$ | C5 | C4 | 123.9(5) |
| O2 | Co1 | O1W | 90.42(13) | C2 | C3 | C2$^{7}$ | 124.2(5) |
| O3$^{2}$ | Co1 | O4$^{1}$ | 85.87(12) | O4 | C4 | C5 | 119.1(4) |
| O3$^{2}$ | Co1 | O4$^{2}$ | 83.93(12) | O4 | C4 | C2 | 123.5(3) |
| O3$^{2}$ | Co1 | O3$^{3}$ | 79.39(3) | C5 | C4 | C2 | 117.4(4) |
| O3$^{2}$ | Co1 | O1W | 97.79(13) | C3 | C2 | C1 | 116.8(3) |
| O1W | Co1 | O3$^{3}$ | 176.57(14) | C3 | C2 | C4 | 118.4(4) |
| Co1$^{4}$ | O4 | Co1$^{5}$ | 94.64(12) | C4 | C2 | C1 | 124.8(3) |
| C4 | O4 | Co1$^{5}$ | 118.6(3) | | | | |

[1]+X,+Y,1+Z; [2]4/3-Y,2/3+X-Y,2/3+Z; [3]2/3+Y-X,4/3-X,1/3+Z; [4]+X,+Y,-1+Z; [5]2/3+Y-X,4/3-X,-2/3+Z; [6]4/3-Y,2/3+X-Y,-1/3+Z; [7]+X,1+X-Y,+Z

## IV. Author Contributions

S. M. V. – data curation, formal analysis, investigation, project administration, writing of original draft - lead
C. G. E. – investigation, writing of original draft - supporting
C. M. R. – investigation, writing of original draft - supporting
S. E. R. – investigation, writing of original draft - supporting
D. N. R. – investigation, formal analysis, writing of original draft - supporting
M. M. – investigation, formal analysis, writing of original draft - supporting
M. R. W. – investigation, formal analysis, validation, writing of original draft - supporting
P. S. W. – data curation, writing of original draft, validation, project administration - supporting
R. E. M. – writing of original draft – supporting; validation, project administration – lead

## V. References

[1] H. Nowell, S. A. Barnett, K. E. Christensen, S. J. Teat, D. R. Allan, *J. Synchrotron Radiat.* **2012**, *19*, 435–441.
[2] G. Winter, *J. Appl. Crystallogr.* **2010**, *43*, 186–190.
[3] G. M. Sheldrick, *Acta Crystallogr. Sect. A: Found. Adv.* **2015**, *71*, 3–8.
[4] G. M. Sheldrick, *Acta Crystallogr. Sect. C: Struct. Chem.* **2015**, *71*, 3–8.
[5] O. V. Dolomanov, L. J. Bourhis, R. J. Gildea, J. A. K. Howard, H. Puschmann, *J. Appl. Crystallogr.* **2009**, *42*, 339–341.
[6] C. F. Macrae, I. Sovago, S. J. Cottrell, P. T. A. Galek, P. McCabe, E. Pidcock, M. Platings, G. P. Shields, J. S. Stevens, M. Towler, P. A. Wood, *J. Appl. Crystallogr.* **2020**, *53*, 226–235.
[7] D. C. Palmer, Crystal Maker Software Ltd, Begbroke, Oxfordshire, England, **2014**.
[8] J. Rouquerol, P. Llewellyn, F. Rouquerol, *Stud. Surf. Sci. Catal.* **2007**, *160*, 49–56.
[9] D. Cattaneo, S. J. Warrender, M. J. Duncan, R. Castledine, N. Parkinson, I. Haley, R. E. Morris, *Dalton Trans.* **2016**, *45*, 618–629.
[10] M. J. Duncan, P. S. Wheatley, E. M. Coghill, S. M. Vornholt, S. J. Warrender, I. L. Megson, R. E. Morris, *Mater. Adv.* **2020**, *1*, 2509–2519.
[11] M. T. Kapelewski, S. J. Geier, M. R. Hudson, D. Stück, J. A. Mason, J. N. Nelson, D. J. Xiao, Z. Hulvey, E. Gilmour, S. A. FitzGerald, M. Head-Gordon, C. M. Brown, J. R. Long, *J. Am. Chem. Soc.* **2014**, *136*, 12119–12129.
[12] J. E. Bachman, M. T. Kapelewski, D. A. Reed, M. I. Gonzalez, J. R. Long, *J. Am. Chem. Soc.* **2017**, *139*, 15363–15370.
